# Pharmacological Phase I Clinical Trials in Pediatric Brain Tumors (1990–2024): A Historical Perspective

**DOI:** 10.32604/or.2025.066260

**Published:** 2025-09-26

**Authors:** Rosa Scarpitta, Emiliano Cappello, Alice Cangialosi, Veronica Gori, Giulia De Luca, Giovanni Gori, Guido Bocci

**Affiliations:** 1 Scuola di Specializzazione di Farmacologia e Tossicologia Clinica, Dipartimento di Ricerca Traslazionale e delle Nuove Tecnologie in Medicina e Chirurgia, University of Pisa, Pisa, 56126, Italy; 2 Azienda Ligure Sanitaria–Alisa, Regione Liguria, Piazza della Vittoria 15, Genova, 16121, Italy; 3 Centro di Farmacologia Clinica per la Sperimentazione dei Farmaci, Azienda Ospedaliera Universitaria Pisana, Pisa, 56126, Italy

**Keywords:** Phase I, clinical trial, pediatric neuro-oncology

## Abstract

Central nervous system (CNS) tumors are the most common solid tumors in pediatric patients and the leading cause of childhood cancer-related mortality. Their rarity compared to adult cancers has made enrolling sufficient cases for clinical trials challenging. Consequently, pediatric CNS tumors were long treated with adult protocols despite distinct biological and clinical characteristics. This review examines key aspects of phase I pediatric oncology trials, including study design, primary outcomes, and pharmacological approaches, along with secondary considerations like clinical responses and ethical aspects. Firstly, we evaluated all phase I trial protocols focusing on pediatric CNS tumors with accessible results published in scientific databases (Pubmed, Scopus, Embase, Web of Science, and Google Scholar) from 1990 to November 2024. Secondly, we searched EudraCT and ClinicalTrials.gov on 30 November 2024 for ongoing trials. Our search yielded 60 completed phase I studies and 15 trials in progress. Dividing them by chronological order revealed that study designs and the response assessments evolved as the understanding of CNS tumor biology increased. Despite advancements improving diagnosis, management, and prognostication, mortality remains high, and morbidity persists. Notably, pediatric pharmacokinetics and pharmacodynamics differ from adults, complicating trial comparisons and dosage optimization. Future efforts should focus on large-scale clinical data collection to enhance trial efficiency.

## 1 Introduction

### 1.1 Pediatric Brain Cancers

Pediatric central nervous system (CNS) tumors are the primary solid neoplasms in the pediatric population, with an incidence rate of approximately 2.9 per 100,000 person-years for malignant tumors. Despite their relative rarity, CNS tumors are the leading cause of childhood cancer deaths in the US, with a mortality rate of around 0.6 per 100,000 person-years [1]. About 7%–8% of these tumors are associated with known brain cancer predisposition syndromes like neurofibromatosis type 1 (NF-1) and tuberous sclerosis or hereditary cancer predisposition syndromes such as Li-Fraumeni syndrome (TP53) and the biallelic mismatch repair deficiency syndrome (bMMRD) [2], while the majority are sporadic with unclear genetic or environmental causes [3].

Due to their paucity compared to adult cancers, classification and treatment approaches of pediatric CNS tumors have been mainly derived from knowledge gained on adult patients. Historically, classifications were based on histologic appearance, but the introduction of comprehensive approaches including next-generation sequencing (NGS), methylome analysis, and proteomics greatly influenced tumor classification, leading to a diagnostic shift from morphology to molecular analyses [4]. Even if they appear to be histologically similar, pediatric tumors differ significantly from those of adults. Pediatric tumors often have mesoderm or neuroectoderm origin, and typically carry a low burden of genetic aberrations, often a single driver event, such as a translocation leading to an oncogenic fusion [5]. Moreover, cancers in children are considered immunologically “cold” tumors since they show very limited immune cell infiltration [6].

For the first time, pediatric tumors have been described in a separate volume in the 2021 World Health Organization (WHO) classification (5th Edition) (WHO CNS5) [7]. General changes in WHO CNS5 on pediatric neuro-oncology have officially recognized pediatric gliomas as distinct entities from adult-type gliomas, ependymomas as categorized based on anatomical compartment, and 15 new tumor types predominantly seen in children and adolescents [8]. Tumor entities frequently diagnosed in pediatric patients are summarized in Table 1.

**Table 1:** 2021 WHO Classification of Tumors of the CNS frequently diagnosed in pediatric patients

CNS tumors	Associated molecular alterations
Gliomas, glioneuronal tumors, and neuronal tumors	
*Pediatric-type diffuse low-grade gliomas*	
Diffuse astrocytoma, MYB- or MYBL1-altered	MYB, MYBL1
Angiocentric glioma	
Polymorphous low-grade neuroepithelial tumor of the young (PLNTY)	BRAF, FGFR family
Diffuse low-grade glioma, MAPK pathway-altered	FGFR1, BRAF, and other MAPK pathway genes
*Pediatric-type diffuse high-grade gliomas*	
Diffuse midline glioma, H3 K27-altered	H3 K27M, H3 K27me3 (loss), EZHIP overexpression, EGFR
Diffuse hemispheric glioma, H3 G34-mutant	H3 G34, TP53, ATRX
Diffuse pediatric-type high-grade glioma, H3-wildtype and IDH-wildtype	IDH-wildtype, H3-wildtype, PDGFRA, MYCN, EGFR
Infant-type hemispheric glioma	NTRK family, ALK, ROS, MET
*Circumscribed astrocytic gliomas*	
Pilocytic astrocytoma	
High-grade astrocytoma with piloid features	Methylation profile, MAPK, CDKN2A, CDKN2B, ATRX
*Ependymal tumors*	
Supratentorial ependymoma	ZFTA, RELA, YAP1, MAML2
ZFTA fusion-positive	
YAP1 fusion-positive	
Posterior fossa ependymomas	H3 K27me3 (loss), EZHIP overexpression
Posterior fossa ependymomas, group PFA	
Posterior fossa ependymomas, group PFB	
Spinal cord ependymal tumors	
Spinal ependymoma	NF2
Spinal ependymoma, MYCN-amplified	MYCN
Myxopapillary ependymoma	–
Subependymoma	–
*Embryonal tumors*	
Medulloblastoma	
Medulloblastomas, histologically defined	
Medulloblastomas, molecularly defined	
Medulloblastoma, WNT-activated	CTNNB1, DDX3X
Medulloblastoma, SHH-activated and TP53-wildtype	PTCH1, SMO, SUFU, GLI2 and EPL1
Medulloblastoma, SHH-activated and TP53-mutant	PTCH1, SMO, SUFU, GLI2 and TP53
Medulloblastoma, non-WNT/non-SHH	MYC or OTX2 amplification, GFI1 or GFI1B overexpression and SNCAIP tandem duplication
*Other CNS embryonal tumors*	
Cribriform neuroepithelial tumor	SMARCB1
CNS neuroblastoma, FOXR2-activated	FOXR2
CNS tumor with BCOR internal tandem duplication	BCOR

Pediatric-type diffuse gliomas mostly lack a clear tumor border on histopathology, and they have been distinguished into low-grade (pLGGs) and high-grade gliomas (pHGGs).

pLGG is a heterogeneous group encompassing tumors of primarily glial or mixed neuronal-glial histology. They represent the most frequently diagnosed brain tumor, accounting for one-third of all pediatric CNS tumor cases [9]. However, the majority of pLGGs have a good prognosis [10], with a 20-year overall survival rate exceeding 80% [11]. According to the new WHO CNS5 classification, 4 tumor types have been identified: (i) diffuse astrocytoma, MYB- or MYBL1-altered; (ii) angiocentric glioma; (iii) polymorphic neuroepithelial tumor of the young; (iv) diffuse low-grade, mitogen-activated protein kinase (MAPK) pathway-altered glioma.

pHGGs are less frequent than pLGGs, with an incidence of 1.1–1.78 per 100,000 children, and account for ∼8%–12% of brain tumors [12]. Despite their low incidence, pHGGs have a poor prognosis, being responsible for over 40% of all childhood brain tumor deaths, with a 2-year overall survival rate of 10%–30%. Four main molecular types have been identified: (i) pediatric-type diffuse high-grade gliomas in diffuse midline glioma, H3 K27-altered, (ii) diffuse hemispheric glioma, H3 G34-mutant, (iii) diffuse pediatric-type high-grade glioma, H3-wildtype and IDH-wildtype, and (iv) infant-type hemispheric glioma. Diffuse pediatric-type high-grade glioma, H3 wildtype and IDH-wildtype, includes approximately one-third to half of pediatric-type diffuse high-grade gliomas [7]. Recent studies identified three major molecular entities, the most common and aggressive of which harbor amplification of MYCN [13].

As their name suggests, circumscribed astrocytic gliomas include gliomas with well-defined borders that separate them from surrounding brain parenchyma. These tumors frequently arise in the posterior fossa, with the cerebellum as the most common site. They share many molecular alterations with pediatric diffuse low-grade gliomas, such as tumorigenesis driven by alterations in the Ras-MAPK pathway. Among circumscribed astrocytic gliomas, pilocytic astrocytomas are the most common CNS tumor in children [14]. Generally, they are low-grade tumors with a favorable prognosis: 5-year OS rate ranges from 80% to greater than 95% [15]. Moreover, in the WHO classification of CNS tumors of 2021, the new tumor entity, high-grade astrocytoma with piloid features, has been proposed as the first CNS tumor defined by a specific DNA methylation profile. Approximately 10% of all cases occur in children [8]. This tumor is seen as having a prognosis only slightly better than IDH wild-type glioblastoma [16].

Pediatric ependymomas are the third most common brain tumors of childhood, after gliomas and medulloblastomas. Ependymomas can occur in all compartments of the CNS; thus, they are now classified based on location, with subtypes based on molecular findings. Ninety percent of pediatric ependymomas are intracranial and, of these, 30% are supratentorial [17]. Two variants of supratentorial ependymoma have been characterized by specific molecular alterations: ZFTA fusion-positive (ST-EPN-ZFTA) and YAP1 fusion-positive (ST-EPN-YAP1) [18]. Prognosis differs between the two variants: the STEPN-ZFTA subgroup comprises higher-risk patients, with a 10-year OS rate of 50% and the ST-EPN-YAP1 subgroup has been reported to have a 100% OS rate in some series [19].

Posterior fossa ependymomas have been subtyped into group A (PFA) and group B (PFB). PFA tends to have an earlier age of onset, a higher incidence of recurrence and mortality than PFB. However, PFB can have late recurrence, with 50% of relapses in one series occurring after 5 years [20].

Spinal cord ependymal tumors comprise four distinct tumor types: spinal ependymoma (SP-EPN), spinal ependymoma with MYCN amplification (SP-MYCN), myxopapillary ependymoma (MPE), and subependymoma (SE). All spinal ependymoma types predominantly occur in adult patients and have a favorable prognosis with 90%–100% survival in 5–10 years [21]; late relapses are frequent [22]. SP-MYCN shows a worse outcome than other spinal ependymomas, and few SP-MYCN cases have been reported in children [23].

Approximately 20% of pediatric brain tumors are embryonal tumors, a heterogeneous group of neoplasms arising from neuroectodermal cells [9]. According to the WHO CNS5 classification, embryonal tumors are primarily categorized into medulloblastomas and other CNS embryonal tumors.

Notably, medulloblastomas account for more than 60% of all embryonal tumors [12]. This type of tumor tends to onset up to 3 years of age and has a rapid growth rate [9]. Symptoms evolve within weeks or months, and typically include increased endocranial pressure, irritability, lethargy, nausea and vomiting, morning headaches, anorexia, and behavioral changes [24]. The CNS5 system now recognizes two types of medulloblastoma: medulloblastoma molecularly defined and medulloblastoma histologically defined. The category medulloblastoma molecularly defined is further divided into four subtypes: (i) WNT-Activated Medulloblastomas (wingless/integrated (WNT)–activated subtype), (ii) SHH-Activated Medulloblastomas (Sonic hedgehog (SHH)–activated medulloblastomas) TP53-wildtype and (iii) SHH-Activated Medulloblastomas TP53-mutant, (iv) Non-WNT/Non-SHH Medulloblastomas [7].

The 2021 5th edition WHO classification sees the category of Embryonal Tumors growing yet again, with inclusion of 3 new genetically defined tumor types: (i) cribriform neuroepithelial tumor (CRINET), (ii) CNS neuroblastoma, FOXR2-activated, and (iii) CNS tumour with BCOR internal tandem duplication [7].

The 2021 5th edition WHO classification further expanded the category of embryonal tumors by including two newly defined genetically characterized tumors: central nervous system neuroblastoma (driven by FOXR2) and CNS tumors with internal tandem duplications of BCOR, alongside the atypical teratoid/rhabdoid tumor (AT/RT) and embryonal tumor with multilayered rosettes (ETMR), already described in the previous classification. Furthermore, the cribriform neuroepithelial tumor (CRINET) was introduced as a distinct entity from AT/RT, a neoplasm primarily occurring in the periventricular regions around 20 months of age. Due to its extreme rarity, the biological behavior of this lesion is not clearly understood [7]. As with other CNS tumors, the general classification of “embryonal CNS tumor NEC or NOS” is included for those embryonal tumors that do not fall into genetically defined categories [25]. Overall, they account for less than 15% of primary brain and spinal cord tumors in children ages 0–14 years [12].

### 1.2 Pharmacological Approaches

As for adult patients, treatment of pediatric CNS tumors involves surgery and a combination of radiotherapy and chemotherapy. Therapeutic strategies differ based on the volume of postoperative residual tumor, the presence or absence of disseminated disease, and the patient’s age, categorizing individuals as average-risk or high-risk. For average-risk patients aged three or older, craniospinal irradiation (CSI) and chemotherapy represent the current recommendation for the post-operative setting. In high-risk cases, treatments involve “standard dose” radiation with concurrent chemotherapy followed by varied maintenance chemotherapy [26].

For many years, traditional chemotherapy has been the cornerstone of CNS cancer therapy in children, and it continues to be crucial and widely used in pediatric cancer therapy [27]. In recent decades, the genetic and molecular characterization of pediatric CNS tumors has led to a radical transformation in the field of pediatric neuro-oncology. Current treatments include targeted therapies, new classes of drugs, and immunotherapeutic approaches [28].

#### 1.2.1 Alkylating Agents

Chemotherapy encompasses a diverse range of compounds, with alkylating agents being a major class of drugs employed in pediatric cancer therapy. Alkylating agents include the nitrogen mustard family, such as ifosfamide and cyclophosphamide, platinum-based agents, and others like temozolomide [27]. These agents exert their cytotoxic effects by forming stable carbocations that create covalent bonds with nucleophilic groups (amino, sulfhydryl, hydroxyl, and phosphate groups) found abundantly in nucleic acids and proteins [29].

Ifosfamide (IFO) is a prodrug used alone or in combination with other drugs to treat various pediatric solid tumors, including rhabdomyosarcoma, soft tissue sarcomas, Wilms’ tumor, bone sarcomas, neuroblastoma, and germ cell tumors [30]. IFO is transformed by the liver enzyme CYP3A4 into 4-hydroxyifosfamide and aldoifosfamide, which then produce isophosphoramide mustard and acrolein [31]. Children’s IFO metabolism varies due to age and prior exposure [32]. IFO has shown improved tumor response rates in phase II trials when combined with etoposide, a podophyllotoxin derivative inhibitor of DNA topoisomerase II, particularly in recurrent or refractory pediatric solid tumors, with response rates ranging from 30%–77% in various tumor types [33]. The highest activity was seen in Wilms’ tumor (77%), Rhabdomyosarcoma (RMS) (69%), germ cell tumors (66%), neuroblastoma (55%), and Ewing’s sarcomas/Primitive neuro-ectodermal tumors (PNETs) (41%–45%) [34].

Platinum compounds are widely used in the treatment of brain tumors. The three major compounds (cisplatin, carboplatin, and oxaliplatin) have a similar pharmacokinetic profile and mechanism of action, but different antitumor activity and toxicity. The cytotoxic effect of platinum compounds depends on their ability to covalently bind to purine DNA bases, thus blocking the proliferation of cancer cells [35]. Unfortunately, various serious side effects have been described for the use of platinum-based chemotherapy, such as nephrotoxicity, neurotoxicity, and ototoxicity [36].

Temozolomide (TMZ) is a second-generation imidazotetrazine derivative that does not require hepatic metabolism to form the cytotoxic methylating agent. TMZ undergoes spontaneous pH-dependent hydrolysis to methyl triazene imidazole-4-carboxamide (MTIC) at a physiological pH. MTIC is then hydrolyzed to the methyldiazonium cation, which is the actual methylating agent of the DNA [37].

Methyldiazonium cation shows its action by converting guanine into O6-methylguanine (O6-MG), N7-methylguanine (N7-MG), and adenine into N3-methyladenine (N3-MA) during DNA replication, resulting in mismatching of base pairs and breaking in DNA double strands, thus inducing the cell cycle arrest at G2/M phase and cell death [38]. In glioma, resistance to TMZ and therapeutic failure are mainly determined by overexpression of O6-methylguanine-DNA methyltransferase (MGMT), an enzyme that plays a key role in reverting the alkylation process done by TMZ [39]. Patients with a methylated MGMT gene promoter benefit from TMZ treatment, achieving a 2-year overall survival (OS) rate of 46% [40]. While approximately 45% of adult high-grade gliomas exhibit MGMT promoter methylation, only about one-quarter of pediatric cases show this alteration. In the adult population, TMZ has demonstrated clinical efficacy, as evidenced by retrospective studies on ependymomas reporting a median progression-free survival (PFS) of 10 months and a median overall survival (OS) ranging from 22 to 30 months [41,42]. By contrast, TMZ has shown limited effectiveness in improving survival outcomes in pediatric patients [43].

#### 1.2.2 Topoisomerase Inhibitors

Camptothecins (CPTs) are a class of natural anticancer drugs derived from the plant *Camptotheca acuminata*, a tree native to China. Since several CPT analogues have been synthesized, only irinotecan and topotecan have been approved for cancer treatment [44]. CPT and CPT analogues act by forming a ternary complex with topoisomerase I and DNA during replication. A collision between the ternary complex and the replication fork results in DNA double-strand breaks and cell death [45]. Interestingly, CPT and its analogues have been demonstrated to have other specific targets affecting cellular protein, RNA, and DNA synthesis [46]. In the context of pediatric primary brain tumors, irinotecan, topotecan, and Karenitecin, a novel highly lipophilic CPT derivative, have demonstrated encouraging results, particularly in high-grade gliomas, medulloblastomas, and ependymomas [47].

#### 1.2.3 Targeted Therapies

The last decades have seen the realization that cellular communication and proliferation pathways in cancer appear to share similar mechanisms that regulate embryonic development. Most interestingly, these pathways, notably including Hedgehog (HH)-Gli, Wnt-βCatenin/Tcf, and Notch, also regulate stem cell self-renewal and survival [48]. Critically, HH-GLI signaling has been implicated in many cancer types, including medulloblastomas and gliomas [49]. Targeting HH-GLI signaling has rapidly driven a general interest in developing novel anti-cancer compounds. Vismodegib (GDC-0449) is the first targeted inhibitor of the Hedgehog (Hh) signaling pathway approved by the U.S. Food and Drug Administration (FDA) [50]. This small molecule is a 2-aryl pyridine that inhibits the Hh pathway by blocking activation of the Smoothened transmembrane protein (SMO), leading to the inhibition of cell cycle proliferation, survival, and differentiation [51]. Although preclinical studies have demonstrated promising results, clinical studies support the efficacy of vismodegib in combination with conventional chemotherapies for the treatment of relapsed or refractory HH-driven medulloblastoma pediatric patients [52] and for a subset of medulloblastoma tumors with PTCH and/or SMO mutations [53].

In pediatric CSN tumors, most driving alterations occur in the MAPK pathway. These kinases are an extensive regulatory network implicated in growth signals and cellular metabolism, thereby affecting cancer progression and therapeutic resistance [54].

BRAFV600E mutation (Class I) and BRAF-fusions (Class II) have been detected in 90% of pediatric low-grade gliomas, whereas mutations in histone H3-encoding genes have been identified in 50% of pediatric high-grade gliomas [55]. Consequently, mitogen-activated protein kinase kinase (MEK) inhibitors (MEKi) and BRAF inhibitors (BRAFi) have been developed, and clinical trials have been designed to assess their efficacy based on a predefined genetic alteration detected in pediatric gliomas [56].

Among MEKi, several recent reports have described Selumetinib (AZD6244, AstraZeneca) to be well tolerated and result in prolonged disease stability in children with progressive LGGs harboring alterations in BRAF or NF1 [57]. However, selumetinib demonstrated limited efficacy in the treatment of pHGG harboring alterations in the MAPK signaling pathway [58].

Among BRAFi, monotherapy with the selective BRAF V600E inhibitor, such as dabrafenib and vemurafenib, has also been shown to have efficacy in children with relapsed or refractory BRAF V600–mutated pLGG [59,60] and pHGG [61]. Activating MAPK pathway mutations are necessary, but not sufficient, to ensure the effectiveness of these treatments in pediatric tumors. Combinations of MAPK inhibitors and additional molecularly targeted, immunotherapeutic, or cytotoxic agents could be required to achieve optimal efficacy in pediatric brain tumors with actionable mutations.

#### 1.2.4 Anti-Angiogenic Agents

Clinical development of humanized monoclonal antibodies (mAb) against vascular endothelial growth factor (VEGF) highlighted the potential of bevacizumab as a possible target therapy for the treatment of HGG, since VEGF is an important stimulus for angiogenesis and possibly tumor invasion. Bevacizumab (Avastin^®^; Genentech) was one of the first angiogenesis inhibitors to be used in clinical settings for the treatment of adult patients with unresectable advanced cancers, usually in combination with chemotherapy [62]. Bevacizumab binds and neutralizes the VEGF, inhibiting the growth of newly formed blood vessels and normalizing tumor vascularization. The safety and good tolerability of bevacizumab have been reported recently in pediatric cancer patients as comparable to adult populations [63–65]. The highest level of efficacy observed in these studies was seen among patients with low-grade CNS tumors [65,66] or in cases with recurrent ependymoma and anaplastic ependymomas harbouring VEGF overexpression [67]. However, the long-term off-treatment benefits of this therapy are not yet well defined [68].

#### 1.2.5 Immunotherapy

Immunotherapy is increasingly used for children with cancer, including CNS tumors. The principle of immunotherapy relies on modulating the immune system’s response to effectively recognize and destroy cancer cells. This goal can be achieved through a wide variety of approaches. The identification of immune checkpoints paved the way to the use of immune checkpoint inhibitors (ICIs) for cancer treatment. In contrast to adults, the pediatric experience has been markedly different, as early studies have shown limited efficacy of ICIs in children. These findings underscore fundamental differences in the immunogenicity of pediatric and adult tumors. The most comprehensive data come from four phase I/II studies published between 2020 and 2022, evaluating nivolumab (ADVL1412; NCT0230445848), pembrolizumab (KEYNOTE-051; NCT0233266849), atezolizumab (iMATRIX; NCT0254160450), and avelumab (NCT0345182551) as monotherapy for refractory or recurrent pediatric tumors. In all four studies, ICIs were well tolerated in children, with weight-based dosing ensuring pharmacokinetics comparable to those observed in adults. However, across these trials, only 3% of patients with solid tumors achieved an objective response. Although many other checkpoints have since been identified and are actively being investigated, unfortunately, they do not seem to provide improved efficacy against tumors with low mutational burden [69].

Cancers with mismatch repair deficiency exhibit a remarkably high rate of mutations, which can cause neoantigen development and increased lymphocyte infiltration. A recent phase 1 trial (NCT02359565) has shown that children with hypermutated high-grade gliomas and mismatch repair deficiency derive clinical benefit from immune checkpoint blockade with a programmed cell death protein 1 (PD-1) inhibitor [70].

The development of chimeric antigen receptor (CAR) expressing T cells is one of the ultimate advances in the field of immunotherapy. CAR-T cells are T lymphocytes genetically modified to express chimeric proteins, known as CARs, targeting selected tumor-associated antigens (TAA) [71]. Currently, the clinical trials reported a strong tumor activity of CAR-T cells directed towards the CD19 antigen in patients with acute lymphoblastic leukemia cells [72,73]. To date, several CAR-T antigenic targets have been considered for pediatric solid tumors [74]. However, due to the heterogeneity in tumor microenvironment and the difficulty of accessing the tumor site, CAR-T cells directed towards a single antigen have not achieved the same sustained success observed in hematological malignancies. For this reason, innovative therapeutic strategies such as next-generation CAR-T cells or combinatory approaches with other immunotherapy agents should be considered for the treatment of pediatric tumors.

In addition to CAR-T therapy, other immunotherapeutic strategies developed in recent decades include therapeutic cancer vaccines, oncolytic virus therapies, cytotoxic T lymphocyte therapies, and engineered T-cell receptors, all currently under investigation as new therapeutic frontiers aimed at improving the prognosis of various pediatric CNS tumors [75].

## 2 Aim

Specifically, our primary objective was to provide a comprehensive description of the historical progression of pediatric oncology phase I trials involving central nervous system tumors from 1990 to 2024. In this context, we focused exclusively on pharmacological therapies while deliberately excluding studies involving non-pharmacological interventions such as radiotherapy.

Accordingly, we undertook to compare study protocols based on the choice of primary outcomes, study design, and evolution of administered drug classes, as well as potential clinical responses to therapeutic approaches. Additionally, our search included ongoing phase I clinical trials to provide an accurate and in-depth picture of the current landscape of pediatric neuro-oncology.

## 3 Methods

We performed two different types of research. Firstly, all relevant literature published from 1990 to November 2024 in prominent databases, including “Pubmed,” “Scopus,” “Embase,” “Web of Science,” and “Google Scholar was systematically gathered using (Phase I OR Clinical Trial OR Study Trial) AND (“oncologic” OR “cancer” OR “tumor” OR “chemotherapy”) AND (“pediatric” OR “paediatric” OR “children” OR “oncopaediatric”) AND (brain OR Central Nervous System OR CNS OR cerebral) as specific keywords. No restrictions on race/ethnicity, geographic area, or language have been applied. Furthermore, reference lists of the retrieved studies have been checked to avoid missing relevant data. All relevant studies have been uploaded to the “Rayyan” website (https://www.rayyan.ai) (Qatar Computing Research Institute), a platform designed for systematic and narrative reviews [76] and screened by titles and abstracts by three pairs of researchers independently and blindly. A predefined set of inclusion criteria has been applied to the selected articles in their full-text version. The inclusion criteria consisted of three main aspects: i) phase I studies, ii) studies involving the pediatric population defined as patients under 25 years of age, and iii) studies exclusively focusing on CNS tumors. We included trials evaluating pharmacological and cellular therapies, but excluded those investigating non-pharmacological interventions such as radiotherapy. We excluded all studies evaluating mixed tumor populations or populations not limited to central nervous system (CNS) tumors, even if CNS tumors were included among the analyzed groups. Only trials that exclusively assessed CNS tumors were included in our analysis. Studies reporting findings in a narrative or non-quantifiable manner, case reports, case series, reviews, letters, conference proceedings, abstracts, and editorials have been excluded. Conflicts or disagreements have been resolved by collegial discussions.

Secondly, EudraCT and ClinicalTrials.gov databases have been interrogated on 30 November 2024 for ongoing phase I clinical trials in pediatric CNS cancer patients. “Central Nervous System Tumors” has been used as a condition/disease keyword, and advanced filters have been applied as described: “Phase I” as Study Trial and “range from 0 to 25 years old” manually entered as age of participants. No restrictions on race/ethnicity, sex, geographic area, or language have been applied.

## 4 Results

**Figure 1:**
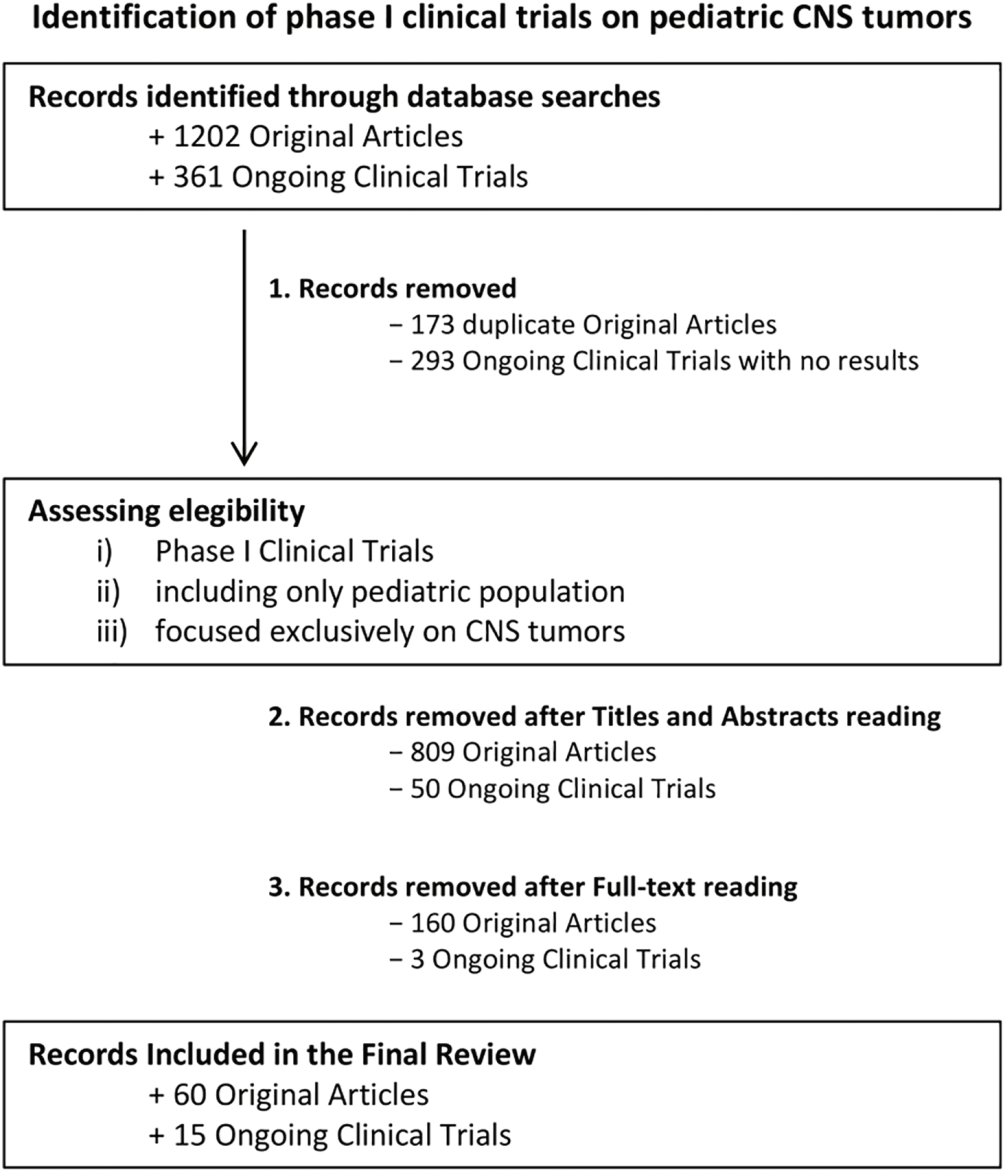
Decision-Making Process for Article Selection in the Final Review. Flowchart illustrates the step-by-step process used to select articles for inclusion in the final review. The process outlines the criteria and stages followed, from initial screening to final inclusion of relevant studies in the analysis. Of the 60 trials identified, 50 (83.3%) were conducted exclusively in the United States. Two trials (3.3%) originated from Canada and two (3.3%) from the Netherlands. Single-country European contributions consisted of one study from Italy (1.7%) and one from France (1.7%). The remaining four trials (6.7%) were multicentre investigations: USA/Canada (n = 1), Germany/Canada (n = 1), France–Italy–Switzerland–United Kingdom (n = 1), and a global consortium involving the United Kingdom, Canada, United States, Australia, France, and India (n = 1). Created using Microsoft PowerPoint (v. 16.98)

In our searches on databases as described above, we identified a total of 1202 studies published from 1990 to November 2024 and 361 ongoing phase I clinical trials on pediatric CNS cancer patients. After removing duplicates and an initial manual screening 220 potentially relevant titles and abstracts have been selected for full-text reading. Following critical analysis, 60 publications have been included and discussed in chronological order to better visualize the progression of clinical trial protocol designs and the selection of primary outcomes as well as the evolution of classes of therapeutics and the potential clinical responses to therapeutic approaches. Furthermore, 15 out of the 361 ongoing clinical trials with published results have been reported in the dedicated paragraph. Selection processes and reasons for exclusion have been reported in the flowchart (Fig. 1).

### 4.1 Phase I Studies from 1990 to 1999

Between November 1990 and March 1993, nine patients under the age of 21, with newly diagnosed high-risk gliomas, were enrolled in a phase I/II study aimed at evaluating the feasibility of a combination of high-dose cyclophosphamide and thiotepa, followed by hyperfractionated radiotherapy and autologous bone marrow transplant. Cyclophosphamide was administered for 4 days at a daily dose of 750 mg/m^2^ via intravenous infusion in the first five patients. For the next two patients, the daily dose was increased to 975 mg/m^2^ but was then reduced back to the initial dose in the final two patients to limit toxicity. Thiotepa was administered intravenously for 3 days at a daily dose of 250 mg/m^2^ in the first three patients and 300 mg/m^2^ for the remaining patients. The patients received a total of 70.2–75.6 Gy, divided into 60 fractions (1.17–1.25 Gy twice daily). Two patients died before receiving radiotherapy. Despite the significant toxicity of this treatment approach, the complete responses lasting over 22 months observed in two patients (one with brainstem glioma and one with anaplastic mixed glioma) were considered highly encouraging. However, overall survival did not improve compared to conventional therapy [77].

In 1993, Pratt et al. tested the efficacy of a 5-day therapy with 2-mercaptoethane sodium sulfonate (Mesna) to reduce the incidence of ifosfamide-induced hemorrhagic cystitis in children with recurrent or progressive primary brain tumors. Patients were grouped into two cohorts based on prior exposure to cisplatin or not and treated with 3 dosage levels of ifosfamide (2133, 2560, and 3072 mg/m^2^) given as a 15-min infusion once every other day for three doses. Mesna was given intravenously at 15 min and 3 and 6 h after initiation of the ifosfamide infusion. Hematologic toxicity was the dose-limiting factor, but prior cisplatin exposure did not increase it. Hyponatremia was the most significant metabolic disturbance, causing seizures in three patients, but it was prevented in subsequent patients by changing the post-ifosfamide hydration fluids from 5% dextrose in quarter normal saline to 5% dextrose in normal saline. Even if no complete responses were observed, authors concluded the need for assessment of ifosfamide with Mesna in a Phase II setting, suggesting up to 3 g/m^2^ of ifosfamide with Mesna given every other day for three doses as recommended dose for a phase II study in children with recurrent brain tumors [78].

In 1995, a phase I/II study assessed the efficacy of a high-dose cyclophosphamide regimen combined with granulocyte-macrophage colony-stimulating factor (GM-CSF) for treating malignant brain tumors. The study enrolled fifteen patients with malignant glioma and six with primitive neuroectodermal tumors (PNET), who received high-dose intravenous cyclophosphamide for two consecutive days (1.8–2.25 g/m^2^ per day; total dose 3.6–4.5 g/m^2^), followed by GM-CSF (5 mg/kg per day) from day 3 to day 11 or until the granulocyte count reached at least 1.5 × 10^9^/L. A total of 83 treatment cycles were administered. The regimen showed efficacy exclusively against PNET, with five of the six patients achieving a partial response. However, the treatment was associated with considerable hematologic toxicity, including severe neutropenia. Additionally, febrile episodes occurred in 54 out of 83 treatment cycles, and one death related to graft-versus-host disease (GVHD) associated with transfusion was reported. In conclusion, while the double-intensity cyclophosphamide regimen demonstrated some activity against PNET, it proved ineffective in treating malignant glioma compared to conventional therapies [79].

In 1998, a single arm Phase I/II study was conducted to improve the survival of children newly diagnosed diffuse pontine gliomas by delivering higher than conventional dose carboplatin with fixed dose of etoposide (120 mg/m^2^/day) [80], a podophyllotoxin derivative that inhibits DNA topoisomerase II. Nine children were treated with a median of six cycles (range 4–10) of chemotherapy during and following 70.2 Gy of hyperfractionated radiation therapy. The carboplatin dose was individually determined to achieve a predetermined area under the plasma concentration × time curve (AUC) using the modified Calvert formula [81]. Subsequently, the carboplatin AUC was escalated by 2 mg/mL × min from 8 to 12 mg/mL × min in successive cohorts of three patients each based on tolerance, using conventional phase I criteria [82]. AUC of 8 mg/mL × min was determined as the maximum tolerated dose (MTD). The treatment was generally well-tolerated with hematologic toxicity being the main concern. Unfortunately, eight of the nine children succumbed to their disease, with a median survival of 44 weeks [80], which was similar to previous studies using radiotherapy alone [83].

**Figure 2:**
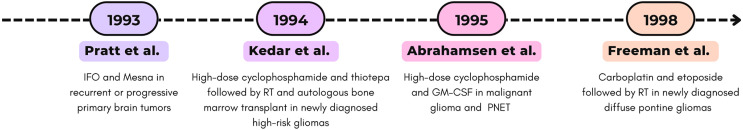
Timeline (1990–1999) of selected Phase I clinical trials. RT, radiotherapy; PNE, Primitive neuroectodermal tumor [77–79,83]

All studies described for the decade 1990–1999 are represented in Fig. 2.

### 4.2 Phase I Studies from 2000 to 2009

The 2000s began with a growing interest in paclitaxel, a representative of a new class of chemotherapeutic agents capable of stabilizing microtubules and preventing their depolymerization. This mechanism interferes with the normal dynamic reorganization of the cytoskeleton, leading to cell cycle arrest [84]. Additionally, it was demonstrated that paclitaxel acts as an effective radiosensitizer, showing significant synergy with radiotherapy in human brain tumors, both *in vitro* [85,86] and *in vivo* [87]. In a short time, paclitaxel became a widely used drug for treating various adult cancers, while results in pediatric solid tumors remained disappointing [88].

In a multicenter phase I study conducted in 2001 by Liu and collaborators, 11 pediatric patients (aged 4–16) with high-grade astrocytomas received concurrent external beam radiation and a unique dose-schedule of paclitaxel, a taxoid antineoplastic agent, as a radiation sensitizer. Paclitaxel was administered intravenously at an initial dose of 1.5 mg/m^2^/24 h as a continuous infusion over 6 weeks, with dose escalation following the standard phase I design. At the 6.5 mg/m^2^/24 h dose, Dose Limiting Toxicity (DLT) was observed in two patients, who required prolonged hospitalization due to severe obstipation. The MTD and the Recommended Phase II Dose (RP2D) were determined to be 4 mg/m^2^/day. For the first time, the authors demonstrated the safety of continuous paclitaxel infusion in combination with radiotherapy in pediatric patients, leaving the evaluation of the potential benefits of taxanes as clinical radiosensitizers for high-grade gliomas to future studies [89].

In the same year, Warren and co-workers published a phase I study aimed to determine the MTD, toxicities, and pharmacokinetics of labradimil combined with carboplatin [90]. Labradimil (Cereport) is a synthetic bradykinin analogue that acts as a potent and specific agonist for the bradykinin B2 receptor [91]. A total of 25 children (≤21 aged) with refractory brain tumours received 4 labradimil dose levels (100, 300, 450 and 600 ng/kg ideal body weight) and carboplatin adaptively dosed to achieve a target plasma AUC of 7.0 mg min/mL over two consecutive days every 28 days. The combination therapy was deemed safe, and the recommended phase II dose for labradimil was 600 ng/kg ideal body weight [90].

In 2002, Hargrave Darren et al. conducted the first-in-child Phase I study using fotemustine, a third generation nitrosourea drug, on refractory paediatric brain tumours. Sixteen ≤21 aged patients with diagnosis of recurrent or resistant primary brain tumour or brain metastases were enrolled. Toxicity and response data were evaluable in 15 out of 16 patients receiving a total of 45 cycles of fotemustine at doses ranging from 100 to 175 mg/m^2^. Main toxicity observed was myelosuppression with 25% of patients requiring dose reduction or delay of subsequent courses. At the dose of 175 mg/m^2^ neutropenia (one of three patients) and grade 4 dose-limiting thrombocytopenia (one of three patients) were recorded. Therefore, a MTD of 150 mg/m^2^ was achieved. Three radiological responses (20% of patients) were found including one partial response and two minor responses, in patients with sarcoma, medulloblastoma and ependymoma, respectively [92].

In 2004, Wagner and collaborators demonstrated that continuous oral treatment with topotecan was well tolerated and somewhat effective in recurrent high-grade glioma. Topotecan was administered orally in ice-cold orange juice at a starting dose of 0.4 mg/m^2^ per day in 32 patients (aged 3–18 years). Hematologic toxicity was the primary side effect observed. The MTD was determined to be 0.9 mg/m^2^ per day. Objective responses were seen in 2 out of 13 evaluable patients, with durations of 2.5 and 9 months [93].

In the following year, Greenberg et al. [94] demonstrated no benefit from combining cyclosporine A [94], a fungal-derived cyclic undecapeptide exhibiting immunosuppressant action [95] with escalating doses of vincristine, an antimitotic Vinca alkaloid [96]. Seven patients (aged 3–21 years) with newly diagnosed diffuse intrinsic brainstem gliomas were treated with a continuous infusion of cyclosporine A and vincristine, etoposide and concomitant radiotherapy. This regimen proved excessively toxic with symptoms ranging from seizures to coma, encephalopathy and hallucinations. Moreover, no changes in the median survival of patients, thus the study was terminated [94].

In 2005 as well, Blaney et al. [97] evaluated the MTD and DLTs of intrathecal (IT) mafosfamide (4-thioethane sulfonic acid salt of 4-hydroxy-cyclophosphamide), a preactivated cyclophosphamide analog [98]. Twenty-five children (≤3 aged) diagnosed with embryonal CNS neoplasm such as medulloblastoma and primitive neuroectodermal tumor were recruited in a standard phase I study. Six dose levels of IT mafosfamide, ranging from 5 mg to 17 mg dissolved in 5 mL of preservative-free saline, were administered according to a 20-week treatment schedule. At the dose of 17 mg, headache and irritability secondary to encephalic pain occurred, so the MTD was 14 mg [97].

The therapeutic efficacy of carboplatin in treating CNS tumors has been extensively demonstrated in phase II studies since the late 1980s [99]. Across all studies, myelosuppression—particularly thrombocytopenia—was identified as the dose-limiting toxicity [81,100,101]. In a phase I study involving adults, carboplatin was administered with autologous bone marrow support, enabling dose escalation up to 2000 mg/m^2^—several times higher than the standard tolerated dose in adults [102].

In 2005, Foreman et al. determined the MTD of carboplatin with peripheral stem-cell rescue in children facing poor-prognosis brain tumors. Twenty children (aged 3–21 years) received a previously determined dose of 3.5 g/m^2^/day of cyclophosphamide for 2 days and carboplatin for 3 days with stem-cell rescue, starting at 400 mg/m^2^/day with increments of 75 mg/m^2^/day in subsequent cohorts. Autologous hematopoietic stem cells were transplanted 48 h after the last dose of carboplatin. Patients at dose level 5 developed grade IV gastrointestinal toxicity, establishing 700 mg/m^2^/day for three days as the MTD. The treatment regimen was well tolerated and resulted in complete responses in 33% of patients and partial responses in an additional 11%, with a median tumor response duration of 10 months and a median follow-up of 35 months (range: 15–87 months) [103].

In 2006, Baruchel et al. assessed the pharmacokinetic profile, MTD and DLT of a continuous oral temozolomide (TMZ) regimen in a phase I study. The study included 27 pediatric patients (≤18 aged) with recurrent or progressive brain tumors refractory to standard therapy. Patients were divided into heavily pretreated (HPT), and not heavily pretreated (NHPT) groups based on prior treatment. Five dose levels of oral TMZ were administered once daily for 42 days, followed by a 7-day rest period for a maximum of 6 cycles. Dose was escalated by 30% if none of patients had DLTs starting from 50 mg/m^2^ for HPT and 75 mg/m^2^ and for NHPT. In both cohorts, all patients had at least one grade 3/4 adverse event included haematological toxicities (thrombocytopenia, lymphopenia, leucopaenia, and anaemia) and non-hematologic effects (vomiting, abdominal pain and anorexia). Over the entire treatment period, 4 patients had a tumor response, while 23 experienced disease progression, with a median PFS of 7.6 weeks and a 1-year PFS rate of 22.2%. Results demonstrated TMZ exhibited linear pharmacokinetics, but with significant inter-patient variability. Authors attribute this variability to the limited sample size in each cohort and various dose levels rather than pharmacokinetic variations as conversion of TMZ to its metabolite is non-enzymatic. Eventually, they encouraged further phase II studies with 85 mg/m^2^ as recommended dose for this 42-day treatment schedule [104].

Meanwhile Gururangan et al. evaluated the effectiveness of intrathecal (IT) Spartaject Busulfan, a water-soluble microcrystalline formulation of dimethanesulfonyloxyalkane, in children with neoplastic meningitis. Responses to the treatment, MTD and DLTs were assessed in a standard phase I trial including 28 children (aged 2–21 years) with leptomeningeal disease from recurrent or progressive primary brain tumors. The starting dose was 5 mg for children >3 years of age and reduced by 20% for younger children. Dose escalation proceeded dosage according to a 5-dose level schedule up to 21 mg. IT Spartaject Busulfan was delivered via an Ommaya reservoir or lumbar puncture twice weekly for 2 weeks followed by an assessment of toxicity and response. Patients with stable disease or objective response received additional IT Spartaject Busulfan and systemic chemotherapy. Chemotherapeutic agents able to penetrate the CSF such as methotrexate, thiotepa, high-dose cytarabine, 5-fluorouracil i.v. or topotecan were not utilized. Overall, IT Spartaject Busulfan was well-tolerated and the recommended dose for subsequent phase II trials was 13 mg. Grade 3 DLTs included headache, neck pain, and chemical arachnoiditis and were observed in 3 patients, one of which received dose level 1 (5 mg) [105].

In 2007, Broniscer et al. determined the MTD and DLTs of escalating doses of TMZ combined with fixed doses of O6-benzylguanine, a pseudosubstrate of MGMT, in a phase I study in children with recurrent brain tumors. Seventy-two patients (≤21 aged) were enrolled and stratified in who had previously received no or local radiotherapy (Str1) and who had undergone craniospinal radiotherapy or myeloablative chemotherapy (Str2). O6-benzylguanine was administered intravenously as a 1-h bolus of 120 mg/m2 followed by 48-h continuous infusion at 30 mg/m^2^/day. Single-dose temozolomide at five dosage levels from 267 to 835 mg/m^2^ was given at least 6 h after completion of O6-benzylguanine bolus. A modified Continual Reassessment Method (CRM) study design was used to estimate the MTDs. The CRM-estimated MTD and the dose-finding MTD in stratum 1 were to 562 and 628 mg/m^2^, respectively. The CRM-estimated MTD and the dose-finding MTD for stratum 2 were 407 and 355 mg/m^2^, respectively. Grade 4 DLTs were predominantly neutropenia, thrombocytopenia and anemia. Four patients completed all planned treatment. Three patients with gliomas had an objective response to therapy, and five patients experienced disease stabilization for at least 6 months. Authors concluded that the combination of temozolomide and O6-benzylguanine was safe, but it demonstrated modest efficacy in patients with recurrent brain tumors [106].

In the same year, Kieran et al. [107] published the first phase I study of the oral lonafarnib (SCH66336, Sarasar; Schering-Plough, Kenilworth, NJ, USA) in pediatric patients with progressive, recurrent CNS tumors. Lonafarnib belongs to the class of farnesyltransferase inhibitors, a group of compounds synthesized to disrupt Ras signaling regardless of its mutational status [108]. Lonafarnib was administered orally twice daily for 28 days at the escalating doses of 70, 90, 115, 150 and 200 mg/m^2^ in 53 children (≤21 aged) with recurrent or progressing CNS tumor diagnoses. DLTs included grade 4 neutropenia at 79 mg/m^2^ (assigned 90 mg/m^2^) and one episode of grade 4 hypokalemia at 118 mg/m^2^ (assigned 115 mg/m^2^). Dose-limiting pneumonitis or myelosuppression was observed in three of three patients at the 200 mg/m^2^/dose level. On the basis of this information, the CRM-estimated MTD was 98.5 mg/m^2^ and the recommended phase II dose resulted 115 mg/m^2^. Of 48 patients assessable for response, 1 patient had a partial response and 9 demonstrated stability of disease. Seven patients remained on therapy for over a year without experiencing disease progression [107].

In 2007, as well, Pollack et al. [109] assessed the MTD and the safety of imatinib, an ATP-competitive inhibitor of the BCR-ABL tyrosine kinase [110] with irradiation in pediatric patients with newly diagnosed brainstem gliomas and recurrent malignant gliomas. Imatinib, also known as imatinib mesylate, STI571, CGP57148B, and Gleevec, disrupts PDGF/PDGFR autocrine and paracrine loops and interferes with the growth of glioma cell lines *in vitro* and *in vivo* [111]. A total of 84 patients (3–21 years aged) with newly diagnosed malignant brainstem gliomas and recurrent malignant gliomas were enrolled. Imatinib was consistently administered twice a day for up to 13 cycles of 28 days each (52 weeks) in the absence of progression or serious toxicity. Newly diagnosed children received imatinib at a starting dose of 200 mg/m^2^, and children with recurrent intracranial malignant gliomas received imatinib at a starting dose of 350 mg/m^2^ with possible escalation to doses up to 800 mg/m^2^ and possible de-escalation to doses of 150 and 100 mg/m^2^. Children with recurrent malignant intracranial gliomas were stratified based on concurrent use of enzyme-inducing anticonvulsant drugs (EIACDs). The CRM-estimated MTD was established only for recurrent high-grade glioma patients not receiving EIACDs at a dose of 541 mg/m^2^, implying a dose-finding MTD of 465 mg/m^2^. Accordingly, this study called attention to a significant rate of hemorrhages. Among 73 patients receiving imatinib, 16 patients experienced symptomatic hemorrhage or asymptomatic hemorrhage detected on the scheduled MRI after beginning therapy. Authors also highlighted the challenges in interpreting issues of toxicity in a tumor type for which the spontaneous rate of a potential adverse event is uncertain [109].

In 2008, MacDonald et al. [112] conducted a phase I trial to determine the MTD and DLT of cilengitide (EMD 121974), an anti-angiogenic small molecule targeting the integrins αvβ3, αvβ5 and α5β1 [113]. Thirty-three <21 years patients diagnosed with refractory CNS tumors received intravenous cilengitide at a starting dose of 120 mg/m^2^ escalating up to a dosage 2400 mg/m^2^. Grade 3 or 4 intratumoral hemorrhage occurred in 13 patients at the dosage level of 2400 mg/m^2^. Therefore, it is concluded that 1800 mg/m^2^ was a safe dose in pediatric brain tumor patients and recommended for phase 2. Finally, one patient with glioblastoma multiforme had a complete response and three patients had stable disease [112].

Based on the promising results observed in preclinical testing [114] and in adult patients [115,116], Gururangan et al. [117] initiated the first pediatric phase I study of alkylating agent cloretazine (VNP40101M; Vion Pharmaceuticals), a sulfonyl hydrazine prodrug with the ability to spontaneously produce nucleophilic species that can effectively alkylate DNA, leading to DNA cross-linking and subsequent cell death [118]. Forty-one children (aged ≤21 years) with recurrent brain tumors were treated with a starting dose of 45 mg/m^2^/d intravenous cloretazine for 5 consecutive days every 6 weeks for up to 8 cycles. Because DLT for this drug was expected to be myelosuppression, patients were divided into two strata based on intensity of prior therapy (moderately pretreated, stratum I; heavily pretreated, stratum II). The starting dose (dose level 1) was the same in both strata while dose escalation and determination of MTD were conducted independently in each stratum. Additionally, de-escalation to dose levels 0 and −1, corresponding to 30 and 20 mg/m^2^/day, respectively, was planned in case dose level 1 proved excessively toxic. As in adults, the primary DLT was myelosuppression, and the MTD in stratum I and II were 45 and 30 mg/m^2^/day for 5 days every 6 weeks, respectively. Three patients showed stable disease for a median duration of 45 weeks after treatment [117].

Haas-Kogan et al. evaluated the MTD, toxicities and preliminary clinical effects of tipifarnib combined to radiation therapy in a traditional phase I dose escalation design in children with newly diagnosed intrinsic diffuse brainstem glioma [119]. Tipifarnib selectively inhibits farnesyltransferase (FTase), an enzyme involved in post-translational modifications of small guanosine triphosphatases like Ras, interrupting Ras mitogenic pathway and other specific signal transduction pathways activated by tyrosine kinase receptors [120]. Seventeen patients (aged 3 to 13 years) received oral tipifarnib at dose levels ranging from 100 to 150 mg/m^2^/dose twice a day for 21 consecutive days in a 28-day cycle, along with conventional fractionated radiation administered once daily for 5 days per week. This was followed by adjuvant tipifarnib at 200 mg/m^2^/dose in a 28-day cycle for up to 24 months in the absence of tumor progression or unacceptable toxicity. The MTD of tipifarnib administered concurrently with radiation was declared as 125 mg/m^2^/dose twice daily. DLTs included skin rash, pneumonia, and neutropenia. One-year survival and one-year PFS estimates were 36.4% and 9.4%, respectively [119].

In 2009, Kieran et al. [121] performed a traditional 3+3 phase I study in children with refractory brain tumors to determine MTD and the safety of semaxinib (SU5416), a small molecule tyrosine kinase inhibitor of the vascular endothelial growth factor receptors 1 and 2 (VEGFR1-2) [122]. Thirty-three patients (aged 1.5–21 years) with brain tumor refractory to standard therapy were enrolled and stratified according to those not receiving enzyme inducing anticonvulsant drugs (EIACD) (Stratum I) or those receiving EIACD (Stratum II). Patients received 110 and 48 mg/m^2^/dose of semaxinib by intravenous infusion twice weekly in stratum I and II, respectively, with planned escalations of approximately 33% increments. Due to serious drug-related toxicities such as grade 3 liver enzyme abnormalities, arthralgia and hallucinations, the trial was closed before completion [121].

**Figure 3:**
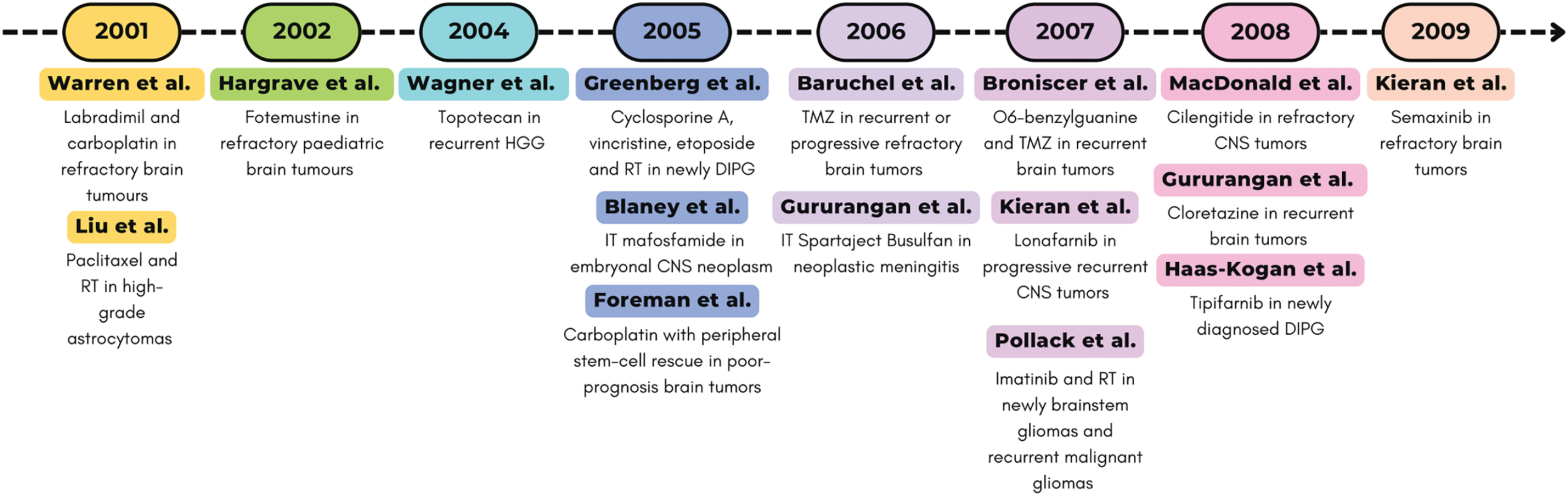
Timeline (2000–2009) of selected phase I clinical trials. Abbreviations: RT, radiotherapy; CNS, central nervous system [89,90,92–94,97,103–107,109,112,117,119,121].

All studies described for the decade 2000–2009 are represented in Fig. 3.

### 4.3 Phase I Studies from 2010 to 2019

In 2010, Ruggiero et al. published the results of a classic 3+3 Phase I study designed to determine the MTD of temozolomide (TMZ) in combination with oral etoposide (VP-16) in 14 pediatric patients (aged 3–28) with progressive or relapsed medulloblastoma. Patients received treatment at dose level 1 (TMZ 120 and VP-16 50 mg/m^2^) and at dose level 2 (TMZ 150 and VP-16 50 mg/m^2^) for a maximum of 12 cycles every 28-days without experiencing dose-limiting toxicities (DLTs). Thrombocytopaenia, anaemia and neutropaenia were observed in patients treated at dose levels 3 (TMZ 150 and VP-16 50 mg/m^2^) and 4 (TMZ 150 and VP-16 50 mg/m^2^). Therefore, TMZ 150 mg/m^2^ on days 1–5 and VP-16 50 mg/m^2^ on days 1–10 was established as the MTD and the recommended dose for TMZ/VP-16 phase II trials. Even several patients had previously received treatments, including single-agent TMZ, intravenous VP-16, and nitrosoureas, one patient achieved a complete response after four cycles, one patient achieved a partial response after one cycle, and seven patients exhibited stable disease after a median of four cycles. These responses to the TMZ/VP-16 combination suggest that the synergy between these two drugs may enhance therapeutic activity compared to their use as single agents [123].

The discovery that Human Epidermal growth factor Receptor 2 (HER2, also name ERBB2), and Receptor tyrosine-protein kinase erbB-4 (ERBB4) are highly expressed in the most aggressive forms of medulloblastoma [124] and ependymomas [125], while Epidermal Growth Factor Receptor (EGFR, also name HER1) is amplified and overexpressed in gliomas [126], has strongly driven the development of inhibitors targeting EGFR and ERBB2 receptors. These include anti-ERBB2 monoclonal antibodies (e.g., trastuzumab [127] and pertuzumab [128]), small-molecule EGFR inhibitors (e.g., erlotinib [129] and gefitinib [130]), and dual EGFR/ERBB2 inhibitors (e.g., lapatinib [131]).

Among EGFR and ERBB2 inhibitors, the pharmacokinetic profile, MTD and DLT of lapatinib were determined by Fouladi et al. in 2010 through a phase I study in children (≤21 aged) with refractory or recurrent CNS malignancies. Lapatinib was administered orally twice daily at escalating doses starting at 300 mg/m^2^ to patients who were not (stratum I; n = 32) or were (stratum II; n = 27) receiving steroids. Rash, diarrhea, and fatigue were identified as DLTs in 6 out of the 50 patients evaluable for toxicity. The recommended phase II dose was 900 mg/m^2^ twice daily, regardless of steroid use. As observed in adults, the maximum plasma concentration of lapatinib and the AUC_0–12_ (area under the curve) increased with dose. However, significant interindividual variability was observed, with an approximately fivefold difference in apparent steady-state oral clearance (ranging from 5.8 to 27.5 L/h/m^2^). Moreover, dose-normalized maximum serum concentration and AUC values were significantly higher in patients from Stratum II compared to those in Stratum I. Additionally, the study revealed high expression of the EGFR family and downstream signaling activation in tumor tissues, particularly in ependymomas, which demonstrated a positive response to lapatinib therapy. Notably, 12 patients (including 5 with ependymomas) maintained stable disease for at least 4 of the 26 treatment cycles. This study demonstrates that lapatinib is well tolerated in pediatric patients and may promote prolonged disease stabilization in some individuals with recurrent malignant central nervous system tumors [132].

In 2011, Geoerger et al. focused on the pharmacokinetic profile, safety and efficacy of erlotinib, another EGFR inhibitor. Fifty children (aged 1–21) with malignant brain tumors received erlotinib in a traditional 3+3 dose-escalation phase I study. Erlotinib was administered orally at 4 dose levels (75, 100, 125, and 150 mg/m^2^) per day in 3-week cycles in monotherapy (Group 1; n = 30) and plus radiotherapy (Group 2; n = 20). A total of 230 treatment-related adverse events were recorded in 44 patients. Most toxicities were grades 1 and 2 dermatological (folliculitis, dry skin, erythema, and pruritis) and gastrointestinal (diarrhea, nausea and abdominal pain) treatment-related adverse events. Twelve patients experienced grades 3–5 treatment-related adverse events, including asthenia, erythema, pruritus, folliculitis, surgical intervention for cyst, interstitial pneumopathy, whitlow, radiodermatitis, and vomiting, intracranial hypertension, intratumoral hemorrhage, neurologic impairment, and seizure with pulmonary aspiration. The mean apparent oral clearance of erlotinib was 4.0 L/h (95% Confidence Interval (CI): 3.4–4.5 L/h), while the mean volume of distribution was 98.6 L (95% CI: 69.8–127.0 L), regardless of the dose level. The mean half-life was 16.6 h. In Group 1, 28% of patients exhibited disease stability, with a median progression-free survival of 1.5 months and a median overall survival of 4.1 months. In Group 2, 17% of patients achieved a partial response after four treatment cycles, while 50% demonstrated disease stability, with a median progression-free survival of 8 months and a median overall survival of 12 months. The 6-month survival rate was 34% in Group 1% and 90% in Group 2. Among patients with brainstem gliomas, those with EGFR overexpression had a median progression-free survival (10.1 months) longer than EGFR-negative cases (6.3 months). The study demonstrated that erlotinib (125 mg/m^2^/day) has an acceptable tolerability profile in pediatric patients with brain tumors and can be safely combined with radiotherapy [133].

In 2011, following preclinical results that demonstrated the antiangiogenic, proapoptotic, and anti-inflammatory activities of lenalidomide [134], Warren et al. conducted a phase I clinical trial in children with recurrent or refractory primary CNS tumors to determine the MTD and assess toxicity and pharmacokinetics. Fifty-one patients, with a median age of 10 years (ranging from 2 to 21 years), received oral lenalidomide for 21 days followed by a 7-day rest. The starting dose of 15 mg/m^2^/day was escalated up to 116 mg/m^2^/day according to a modified continuous dosing schedule. As in adults, myelosuppression was the main side effect, but doses up to 116 mg/m^2^/day were well-tolerated; thus, the MTD was not defined. Objective responses were observed in two patients at the highest dose levels (88 and 116 mg/m^2^/day). However, the authors concluded that, despite the incomplete understanding of its antitumor mechanisms, lenalidomide appears to have activity in patients with recurrent, refractory, and progressive CNS tumors, although long-term toxicity may be a limiting factor [135].

In 2013, Kilburn et al. [136] conducted a phase I study using a 3+3 dose-escalation design to determine the MTD and DLT of capecitabine, an oral fluoropyrimidine carbamate converted to 5-fluorouracil (5-FU) by thymidine phosphorylase, an enzyme preferentially expressed in various tumor types [137]. Capecitabine was administered in rapidly disintegrating tablets of 125, 175, 250, and 350 mg. Twenty-four patients (ages 3–21) with newly diagnosed brainstem gliomas and high-grade gliomas received capecitabine twice daily without interruption for 9 weeks, beginning within 24 h of starting RT. The initial dose was 500 mg/m^2^ administered twice daily, with dose increments of 30% for each subsequent level. After a 2-week break, patients received a maintenance dose of 1250 mg/m^2^ (2500 mg/m^2^/day) for 14 consecutive days, followed by a 7-day break, for a total of three post-radiation courses. The full protocol treatment lasted 20 weeks. DLTs were observed at dose levels 2 (650 mg/m^2^) and 3 (850 mg/m^2^), including palmar-plantar erythrodysesthesia (grade 2 [n = 1] and grade 3 [n = 1]) and a grade 2 ALT elevation that did not resolve to baseline within 7 days. Based on these findings, the MTD and recommended phase II dose of capecitabine administered concurrently with RT was established at 650 mg/m^2^ every 12 h [136].

In 2013, Hummel et al. [138] published a phase I trial evaluating the use of vorinostat, an oral histone deacetylase inhibitor [139], in combination with temozolomide for treating refractory or recurrent CNS malignancies. The study included nineteen patients, aged 1 to 21 years, with refractory or recurrent primary brain or spinal cord tumors for which no known curative therapy was available. Vorinostat, followed by 150 mg/m^2^/day of temozolomide approximately one hour later, was administered orally once daily for 5 consecutive days every 28 days across 3 dose levels using the rolling 6 design. The starting dose of vorinostat, 230 mg/m^2^/day (dose level 1), was increased to 300 mg/m^2^/day at dose level 2, while temozolomide remained at 150 mg/m^2^/day for both levels. At dose level 3, vorinostat remained at 300 mg/m^2^/day, while temozolomide was increased to 200 mg/m^2^/day. Each treatment cycle lasted 28 days and could be repeated up to 13 times. The distribution of vorinostat, administered in combination with temozolomide, was similar to that observed in other pediatric studies [140] and in adults receiving vorinostat monotherapy [141]. A considerable variability was observed in the pharmacokinetics of vorinostat and its inactive metabolites (4-anilino-4-oxobutanoic acid and VOR-glucuronide) at each dose level, preventing the establishment of a clear dose-exposure relationship across the two evaluated dose levels. Overall, the combination was well tolerated, resulting in a partial response in one patient and stable disease in three patients. Myelosuppression was reported as DLT in 4 patients at dose level 3, thus defining the pediatric MTD and RP2D for the combination of vorinostat and temozolomide as 300 and 150 mg/m^2^/day, respectively. Stable disease and partial response were observed in three patients and one patient, respectively [138].

In 2013, Gajjar et al. [142] designed a phase I trial to determine the toxicity, pharmacokinetics, and recommended phase II dosage of the smoothened inhibitor vismodegib [50] in pediatric patients with refractory or recurrent medulloblastoma. Thirteen children (aged 3–21 years) received either 85 or 170 mg/m^2^/day for 28 days. During enrollment, the 25 mg capsules were no longer available, so they revised the protocol. To minimize deviation from the target dose of 170 mg/m^2^/day, they adopted a flat-dosing strategy. In the revised study protocol, a flat-dosing scheme was used: smaller patients (Body Surface Area, BSA 0.67–1.32 m^2^) received 150 mg/day, and larger patients (BSA 1.33–2.2 m^2^) received 300 mg/day. Only three patients experienced DLTs (grade 3 γ-glutamyl transferase elevation, grade 4 hypokalemia, and grade 3 thrombocytopenia), so they concluded that treatment with vismodegib is safe and feasible in pediatric patients. Vismodegib demonstrated pharmacokinetics in children comparable to those in adults. Consistent with responses seen in adults, antitumor activity was observed in 1 of 3 patients with SHH-subtype disease whose tumors were evaluable, while no responses were observed in patients from other subgroups [142].

The same year, Broniscer et al. [143] conducted the first phase I trial using a combination of small-molecule inhibitors in children with newly diagnosed diffuse intrinsic pontine glioma (DIPG). Since the VEGF and PDGF pathways are critical in gliomas, they selected vandetanib, an inhibitor of both VEGFR-2 and EGFR [144] and dasatinib, an inhibitor of PDGFR [145] Twenty-five children (aged 18 months to 20 years) with newly diagnosed DIPG were enrolled to evaluate the safety, MTD, pharmacokinetics, and pharmacodynamics of this combination administered during and after radiotherapy. Four dose levels were initially planned, with escalating doses of vandetanib (65, 85, and 110 mg/m^2^ once daily) and dasatinib (65 and 85 mg/m^2^ per dose, twice daily). Unlike the traditional design, six evaluable patients were required to be treated at each dose level unless the dose level was deemed too toxic. Lower dose levels were added during the study once the starting dose level was found to be too toxic. The treatment was well tolerated overall, with toxicities including intolerable diarrhea and significant myelosuppression. Plasma pharmacokinetics studies showed that the steady-state exposure and C_max_ of vandetanib in children were slightly higher than those observed in adults, while the steady-state exposure of dasatinib was similar to that seen in adults. Although the combination of RT, vandetanib, and dasatinib did not improve the poor prognosis for children with DIPG, the study provided valuable insights into the safety and pharmacokinetics of these drugs in pediatric patients [143].

Also in 2013, Cohen et al. [146] presented a phase I trial evaluating the safety and tolerability of arsenic trioxide (ATO), a chemotherapeutic agent [147], when administered concurrently with radiation therapy. Twenty-four children with newly diagnosed anaplastic astrocytoma, glioblastoma, or DIPG received radiotherapy at dosages ranging from 5400 to 5940 cGy (depending on tumor type and location) over approximately 6 weeks in all cases. Dose level 1 involved administration of 0.15 mg/kg of ATO once per week, with planned dose escalation up to dose level 5, which involved daily ATO administration on all weekdays during radiation therapy. The study was amended following a report by Fox et al., recommending a daily dose of 0.15 mg/kg of ATO due to dose-limiting Corrected QT interval prolongation or pancreatitis when the dosage was increased to 0.2 mg/kg/day in children with leukemia [148]. The therapy was well tolerated, with mild toxicities and no significant cardiac toxicities. The median overall survival was 10 months (range 2–22) for DIPG cases, 9 months (range 8–23) for anaplastic astrocytoma cases, and 13 months (range 11–33) for glioblastoma multiforme cases [146].

In 2014, Su et al. [149] conducted a traditional 3+3 phase I dose-escalation trial to evaluate the MTD, DLTs and pharmacokinetics of veliparib—an oral PARP inhibitor [150]—combined with TMZ in 31 children under 21 years old with refractory or recurrent primary CNS malignancies for whom no curative therapy was available. Veliparib was administered twice daily and TMZ once daily on days 1–5 of each 28-day cycle. The starting doses were 20 mg/m^2^ b.i.d. for veliparib and 180 mg/m^2^/d for TMZ. Planned dose escalations increased veliparib in 5 mg/m^2^ increments up to a maximum of 30 mg/m^2^ per dose b.i.d., followed by a single TMZ escalation to 200 mg/m^2^/d. Due to higher-than-expected rates of myelosuppression at the first two dose levels, reductions in the TMZ dose were required. Veliparib concentrations and poly (ADP-ribose) (PAR) levels in Peripheral Blood Mononuclear Cells (PBMC) were measured on days 1 and 4. Analysis of pharmacokinetic and PBMC PAR levels was performed twice during study to rationally guide dose modifications and to determine biologically optimal MTD/RP2D. The resultant data suggested veliparib 25 mg/m^2^/dose b.i.d. and TMZ 135 mg/m^2^/d for 5 days every 28 days were declared the RP2Ds for this regimen. Only 2 out of 12 patients treated at RP2Ds experienced dose-limiting toxicities. At the RP2D of veliparib, pediatric pharmacokinetic parameters were similar to those in adults. Although no objective response was observed, 14 patients experienced stable disease (SD) lasting at least 8 weeks, with 4 patients having SD for over 6 months, including 1 with glioblastoma multiforme and 1 with ependymoma [149].

Piha-Paul et al. [151] published a Phase I, open-label study of temsirolimus, an mammalian target of rapamycin (mTOR) inhibitor [152], in combination with bevacizumab in pediatric patients with CNS tumors. The study aimed to assess the safety, tolerability, and efficacy of this therapeutic combination; therefore, only six patients (aged 3–14 years) with malignant CNS tumors refractory to standard-of-care therapies (following 2–3 prior systemic treatments) were enrolled. Dose escalation in the trial successfully reached dose level 13, representing the highest FDA-approved doses of both drugs (bevacizumab 15 mg/kg i.v. every three weeks and temsirolimus 25 mg i.v. weekly) [153]. Temsirolimus was administered at a dose of 25 mg i.v. on days 1, 8, and 15, while bevacizumab was administered at doses of 5, 10, or 15 mg/kg on day 1 of each 21-day cycle, continuing until either disease progression or patient withdrawal. No patients experienced grade 4 toxicities. Grade 3 toxicities potentially related to the study drugs occurred in two patients: one exhibited anorexia, nausea, and weight loss, and the other experienced thrombocytopenia and elevated alanine aminotransferase levels. All other adverse effects were grade 2 or lower. No dose reductions were required for either bevacizumab or temsirolimus due to toxicity. A partial response was observed in one patient, with a 51% reduction in tumor size after four cycles of therapy in a patient with GBM. Disease stabilization for a median duration of 16 weeks (range 8–47 weeks) was achieved in four additional patients (one each with GBM, medulloblastoma, ependymoma, and pontine glioma). Overall, this combination therapy was well tolerated and demonstrated potential efficacy in the treatment of pediatric CNS tumors [151].

In 2015, Kilburn et al. [154] published a study evaluating the MTD and toxicity of enzastaurin, a potent oral serine/threonine kinase inhibitor targeting protein kinase Cβ (PKCβ) and the phosphoinositide 3-kinase (PI3K)/ Akt pathways [155,156], in 33 patients under 22 years of age with histologically confirmed recurrent, progressive, or refractory primary CNS tumors. Enzastaurin was administered orally once daily in 28-day cycles. The starting dose was 260 mg/m^2^/day, with escalation to 340 and 440 mg/m^2^. The 440 mg/m^2^ dose level was expanded to further evaluate the toxicity and pharmacokinetics of both once-daily (7 participants) and twice-daily dosing (220 mg/m^2^ b.i.d.) in 14 participants. Enzastaurin was generally well tolerated, with no grade 4 toxicities reported. All observed toxicities were grade 2 or lower, except for one participant who experienced grade 3 lymphopenia among the 10 evaluable participants in the dose-escalation phase or the expanded cohort of 7 participants receiving once-daily dosing at the maximum dose level of 440 mg/m^2^/day. With twice-daily dosing at 440 mg/m^2^/day, 2 of the 12 evaluable participants experienced grade 3 thrombocytopenia and grade 3 ALT elevation. This study was the first to evaluate enzastaurin in children, establishing the recommended phase 2 dose for enzastaurin in children as 440 mg/m^2^/day once daily. Although well tolerated, no objective responses were observed; however, 11 participants achieved stable disease for more than 3 cycles, with a median time on therapy of 1.9 months (range, 0.4–17.5 months). The authors suggested that future clinical trials of enzastaurin in children should combine it with radiotherapy, antiangiogenic agents, or traditional chemotherapy [154].

In the same year, Packer et al. [157] conducted a Phase I study to evaluate the MTD, DLT, and pharmacokinetics of emvododstat (PTC299), an oral small molecule that selectively inhibits VEGFR at the post-transcriptional level [158]. Twenty-seven pediatric patients (ages 3–21 years) with recurrent CNS tumors received oral emvododstat following a rolling-six design. The initial dose was 1.2 mg/kg administered twice daily, with escalation to 2 mg/kg administered three times daily. The pharmacokinetics of PTC299 showed a dose-proportional increase in exposure. Steady-state drug exposures suggested accumulation with multiple administrations over time. Furthermore, the median AUC0–Tlast values at a dose of 2 mg/kg (7.659 ng/mL at 9 h) were similar to the exposures observed in adult studies. The therapy was well tolerated at the highest dose level tested (2 mg/kg/dose TID). Grade 3 hyponatremia in one patient was the only dose-limiting toxicity (DLT) observed. The study was terminated while patients were receiving the highest planned dose due to hepatotoxicity observed in ongoing phase I studies in adults. At the time of study closure in February 2012, three patients were receiving the 8th, 13th, and 18th doses of treatment. No complete or partial responses were observed. Two patients with low-grade gliomas showed an objective tumor reduction (greater than 25% but less than 50%). Three other patients remained on treatment for over seven cycles before experiencing disease progression [157].

In 2015, Chastagner et al. [159] conducted a Phase I study to determine the MTD and pharmacokinetics of non-pegylated liposomal doxorubicin (Myocet^®^), a novel chemotherapeutic delivery system [160], in children with recurrent or refractory high-grade glioma. Liposomal encapsulation of doxorubicin has been shown to both reduce toxicities [161,162] and improve doxorubicin delivery to brain tumors [163]. Thirteen patients aged 6–17 years, refractory to previous chemotherapy and radiotherapy, received intravenous Myocet^®^ over a 1-h infusion on day 1 of a 21-day cycle. The starting dose of 60 mg/m^2^ was escalated to the adult recommended dose (RD) of 75 mg/m^2^. No DLTs occurred in the seven patients treated at the starting dose level. At the 75 mg/m^2^ dose, two out of six patients experienced grade 3–4 toxicities across all cycles, including neutropenia, thrombocytopenia, vomiting, nausea, mucositis, and fever. Consequently, the MTD and RD for the Myocet^®^ were established as 60 mg/m^2^, administered as a 1-h infusion on day 1 of a 21-day cycle. The volume of distribution at steady state, clearance, and elimination half-life of doxorubicin were estimated to be 24.8 L, 15 L/h/m^2^, and 34.8 h, respectively. However, significant interpatient variability and a lack of dose proportionality between the two dose levels were observed. Treatment was discontinued due to disease progression in 12 out of 13 patients. The authors recommended further studies to assess the efficacy of Myocet^®^ in pediatric patients with high-grade glioma in Phase II trials [159].

In the same year, Kieran et al. [164] published the evaluation of cediranib (AZD2171), an oral pan-vascular endothelial growth factor (VEGF) receptor tyrosine kinase inhibitor [165], in children and adolescents less than 22 years of age with recurrent or refractory primary CNS tumors. The study aimed to determine its toxicity profile, DLTs, MTD, PK, and pharmacodynamics. Thirty-six patients in stratum I and 12 patients in stratum II were initially assessed. An MTD of 32 mg/m^2^/day was declared, but excessive toxicities, including Grade 3 dehydration, confusion, ocular/visual issues, pain, palmar-plantar erythrodysesthesia syndrome, elevation in transaminases (ALT-SGPT, AST-SGOT), fatigue (lethargy, malaise, asthenia), and Grade 2 proteinuria, suggested that it might not be tolerated over a longer time period. At 20 mg/m^2^/day, steady-state cediranib exposure in children (239 ng/mL·h) matched that of a 5 mg flat dose in adults (226 ng/mL·h), indicating potential age-related differences in drug sensitivity. These data suggest that children and adolescents may be less susceptible to the pharmacological effects of cediranib, both in terms of toxicity and antitumor efficacy, due to lower systemic exposure compared to adults at equivalent doses. Two partial responses were recorded, with a tumor volume reduction of more than 50%, sustained for at least 6 weeks. Additionally, a patient with recurrent high-grade glioma, who had started therapy after a complete macroscopic resection, completed two years of treatment with cediranib in continuous complete remission [164].

Also in 2015, Hoffman et al. [166] conducted a phase I trial where children (aged 3–21 years) with recurrent CNS malignancies were treated with MK-0752, an oral gamma-secretase inhibitor that targets the Notch signaling pathway by inhibiting γ-secretase, blocking Notch1 signaling [167]. MK-0752 was administered once weekly to 10 patients with various CNS tumor types at two dose levels: 1000 and 1400 mg/m^2^. Fatigue was the DLTs observed in one case at 1000 mg/m^2^ dosage, while no DLTs occurred at 1400 mg/m^2^. Grade 3 toxicities included lymphopenia, neutropenia, and anemia. NOTCH protein levels decreased in six out of seven patients 24 h after dosing, indicating effective target inhibition. The drug achieved sufficient blood concentrations to support this effect, with peak levels reaching 88.2μg/mL at the 1000 mg/m^2^ dose and 60.3 μg/mL at the 1400 mg/m^2^ dose. No objective responses were observed, but two out of nine patients achieved prolonged disease stabilization for 6 and 10 treatment cycles. The study was prematurely terminated due to the withdrawal of support from the pharmaceutical company. However, the results demonstrate that MK-0752 was well-tolerated at the highest dose tested (1400 mg/m^2^ weekly), and target inhibition in PBMCs was consistently observed, suggesting the attainment of a biologically effective dose [166].

In 2016, Mitchell et al. [168] presented the results of a phase I study conducted on 18 pediatric patients with refractory or relapsed neuroblastoma or medulloblastoma, aimed at determining the safety and tolerability of TPI-287 alone or in combination with TMZ. This was the first study of TPI-287, a third-generation synthetic taxane capable of crossing the blood-brain barrier [169]. Although it had been previously studied in adult populations, this study represents the first evaluation of the drug in the pediatric setting. The drug was administered intravenously on days 1, 8, and 15 of each 28-day cycle, starting with an initial dose of 90 mg/m^2^ and escalating by 20–25 mg/m^2^ per cohort until the MTD was determined according to a 3+3 study design. Patients received TPI-287 as monotherapy for the first two cycles, followed by the same administration schedule in combination with oral TMZ (100 mg/m^2^/day on days 1–5) starting from cycle 3. Patients with disease progression during cycles 1 or 2 were allowed to move directly to cycle 3. The treatment was well tolerated. Dose-limiting toxicities included hemorrhagic cystitis and sensory neuropathy at 135 mg/m^2^, while the most common non-dose-limiting toxicities were anorexia and pain. As in adults, the MTD of TPI-287 was established at 125 mg/m^2^ administered intravenously on days 1, 8, and 15 of a 28-day cycle, both as monotherapy and in combination with TMZ. The pharmacokinetic analysis of TPI-287 at the 125 mg/m^2^ dose showed a C_max_ (3.2 mg/mL), similar to that in adults at the 126 mg/m^2^ dose (3.4 mg/mL), but a lower AUC (5.10 mg/mL·h) compared to adults (6.1 mg/mL·h). This is likely due to a shorter half-life (2.7 h) in pediatric patients compared to adults (9.26 h). These data indicate a faster drug clearance in children, suggesting that in the pediatric population, TPI-287 dosing may require more frequent administration [168].

In 2017, Banerjee et al. [170] published the result of a phase I study to assess the dose-limiting toxicities and antitumor activity of selumetinib, a MEK inhibitor [171], in children with progressive low-grade gliomas (LGGs). Thirty-eight eligible subjects, aged 3 to 21 years, were enrolled. In the initial phase, the treatment was administered to patients aged ≥12 years at a starting dose of 33 mg/m^2^/dose b.i.d., with planned dose escalations up to 95 mg/m^2^/dose b.i.d., and dose reductions to level 0 (25 mg/m^2^/dose b.i.d.) in the case of toxicity. Since 4 out of 11 patients treated at dose level 1 experienced dose-limiting toxicities (DLT), including increased amylase/lipase, rash, and mucositis, this dose was considered excessively toxic. The protocol was subsequently modified to include dose levels −1 (20 mg/m^2^/dose b.i.d.) and −2 (15 mg/m^2^/dose b.i.d.), allowing for further dose reduction and the inclusion of patients aged 3 to 12 years. None of the 3 patients aged ≥12 years treated with the reduced dose of 25 mg/m^2^/dose b.i.d. experienced DLT, establishing this dose as the recommended phase 2 dose (RP2D). In the expansion cohort, 3 out of 12 patients aged ≥12 years experienced DLT, while none of the 12 patients aged <12 years developed DLT, confirming 25 mg/m^2^/dose b.i.d. as the RP2D for both cohorts. The median AUC and apparent oral clearance of selumetinib at the RP2D were 3855 ngh/mL (range: 1780–7250 ngh/mL). Thirteen of the 19 tumors analyzed had BRAF alterations, with 4 of these (2 with the BRAF-KIAA1549 fusion, 1 with the BRAFV600E mutation, and 1 with both alterations) showing prolonged partial responses at the RP2D. Patients received a median of 13 treatment cycles, and 14 completed the protocol with at least one instance of disease stabilization. The 2-year progression-free survival at the RP2D was 69% [170].

Veldhuijzen van Zanten et al. [172] investigated the safety, tolerability, and preliminary efficacy of adding gemcitabine, an antimetabolite (deoxycytidine analog) [173], as a radiosensitizer in children aged 3–18 years with newly diagnosed DIPG. Gemcitabine was administered to 9 patients intravenously weekly for six doses, concomitantly with six weeks of radiotherapy, using a dose-escalation schedule (140, 175 and 200 mg/m^2^) without an expansion cohort. No dose-limiting toxicities were observed up to the maximum dose of 200 mg/m^2^, and all patients experienced symptom reduction. Quality of life tended to improve during treatment. Progression-free survival (PFS) and median overall survival (mOS) were 4.8 months (95% CI 4.0–5.7) and 8.7 months (95% CI 7.0–10.4), respectively [172].

Also in 2017, Becher et al. [174] conducted a phase I study utilizing a standard 3+3 dose-escalation to evaluate MTD, PK and toxicities of the Akt inhibitor perifosine [175]. Twenty-three paediatric patients (age 4–18) with various types of CNS tumors received a loading dose on day 1, followed by subsequent maintenance doses every 1–4 days, depending on the dose level and body surface area, with the aim of reaching dose levels of 25, 50, 75, 100, and 125 mg/m^2^ per day. The treatment was well tolerated, and perifosine was found to be safe. Only one dose-limiting toxic effect occurred, namely grade 4 hyperuricemia at dose level 4. Neutropenia was the most common grade 3 or 4 toxicity (8.7%). Steady-state plasma levels of perifosine showed saturable dose exposure at 50 mg/m^2^/day, with significant inter-patient variability. Although levels were generally higher in pediatric patients compared to adults, the MTD was not defined, and the recommended dose for phase II was found to be 50 mg/m^2^/day. Despite some patients maintaining stable disease for two months (one patient with DIPG at dose level 5 and four patients with high-grade glioma at dose levels 2 and 3), no objective responses were observed [174].

In 2018, Broniscer et al. [176] evaluated the safety and tolerability of dasatinib, a PDGFRA inhibitor [152], in combination with crizotinib, a c-Met inhibitor [177], in patients aged 2 to 21 years with HGG (n = 14) and DIPG (n = 11). Patients received two different therapeutic regimens: a twice-daily administration of dasatinib at 50 mg/m^2^ and crizotinib at 130 or 100 mg/m^2^ for a 6-week cycle, and a once-daily administration of dasatinib at 50 mg/m^2^ and crizotinib at 165 mg/m^2^ for a 28-day cycle up to 2 years. The combination of dasatinib at 50 mg/m^2^ and crizotinib at 130 or 100 mg/m^2^, administered twice daily, was poorly tolerated. However, when the regimen was adjusted to once-daily dosing with reduced doses, a tolerable MTD was identified (dasatinib at 50 mg/m^2^ and crizotinib at 215 mg/m^2^). Grades 3 and 4 DLTs such as diarrhea, fatigue, proteinuria, hyponatremia, rash, and neutropenia were observed, with no evidence of significant clinical responses. The pharmacokinetic parameters of both drugs were consistent with previous studies. Based on these findings, the authors discouraged further testing of this drug combination in children due to poor tolerability and limited efficacy [176].

**Figure 4:**
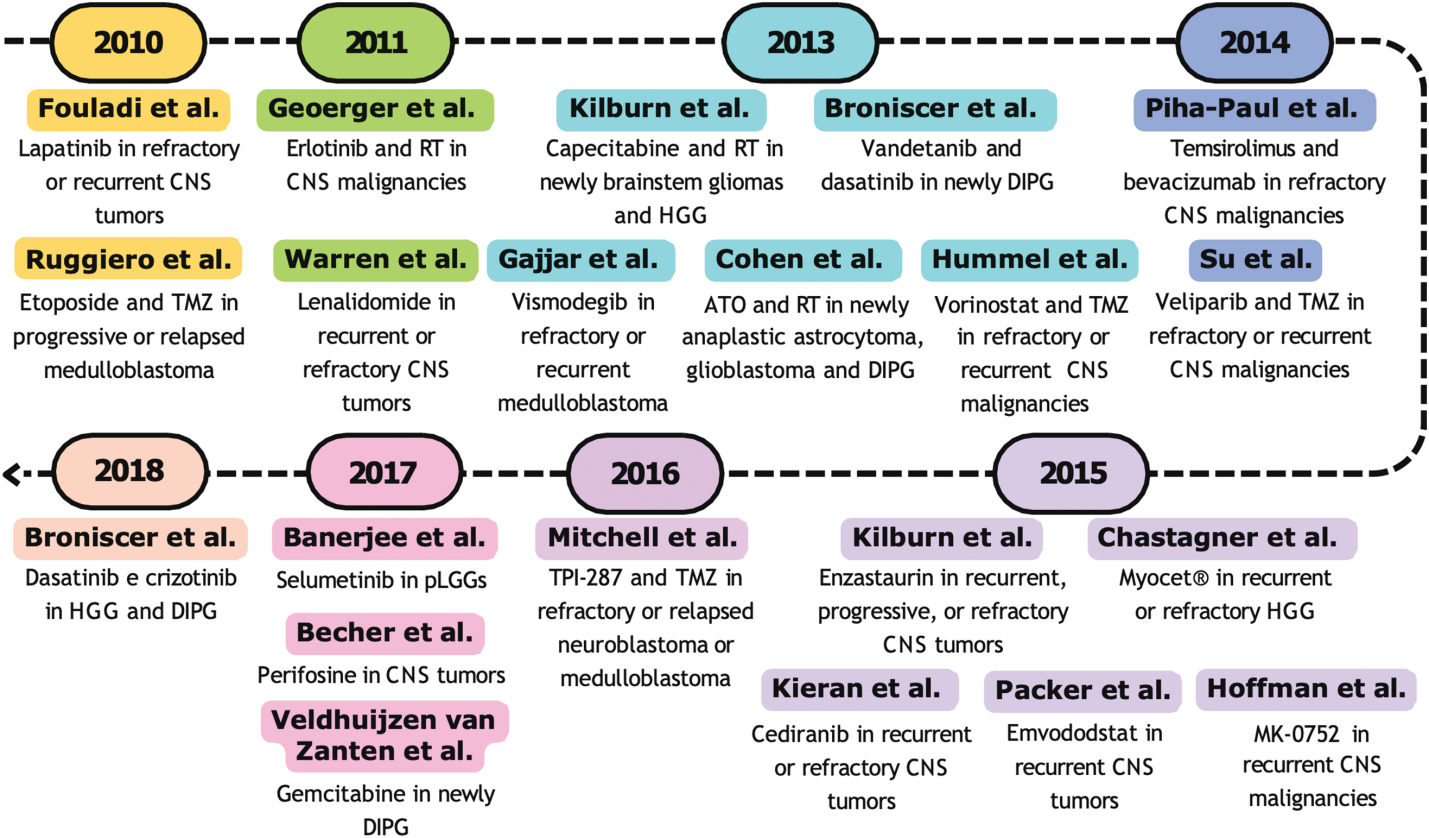
Timeline (2010–2019) of selected phase I clinical trials. RT, radiotherapy; TMZ, temozolomide; CNS, central nervous system; DIPG, diffuse intrinsic pontine gliomas; pLGG, pediatric low-grade gliomas [123,132,133,135,136,138,142,143,146,149,151,154,157,159,164,166,168,170,172,174,176]

All studies described for the decade 2010–2019 are represented in Fig. 4.

### 4.4 Phase I Studies from 2020 to 2024

The last decade began with the phase I study by Hipp et al., [178] the first to evaluate lenalidomide, a potent thalidomide analogue with immunostimulatory, anti-inflammatory, anti-angiogenic, and pro-apoptotic properties, in combination with radiotherapy in pediatric patients with newly DIPG or High-Grade Glioma (HGG). Building on the results obtained from the pediatric clinical trial conducted by Warren et al. in 2011 [135], Hipp et al. designed a phase I study (NCT01222754) to determine the tolerability and toxicity profile of lenalidomide. The dose-escalation phase involved twenty-nine patients aged 4 to 19 years, who received lenalidomide at an initial dose of 10 mg/m^2^ per day, with increments up to 116 mg/m^2^ per day, in combination with standard doses of radiotherapy (1.8 Gy/daily fraction for a total dose between 54 and 59 Gy) over a 6-week period. In the first three dose levels (32, 52, and 88 mg/m^2^ per day), no dose-limiting toxicities (DLTs) were observed. Two of the 14 patients treated at the highest dose developed grade 3 hemolytic anemia and grade 4 thrombocytopenia, which required treatment discontinuation. As in the study by Warren et al. [135], the MTD was not reached, and the dose of 116 mg/m^2^ per day was identified as the RP2D. Following the dose determination phase, patients underwent a 2-week break, followed by a maintenance phase, during which they received lenalidomide at the RP2D for 21 days in a 28-day cycle. In the absence of unacceptable toxicity or disease progression, treatment could be repeated for up to 24 cycles. Each patient received a median of 7 treatment cycles (range: 1–41). Nine patients required dose reductions between the 3rd and 6th cycles, while two patients had to discontinue treatment due to myelosuppression. The median PFS and OS in the DIPG cohort were 6.5 months (range: 126–1166 days) and 11.1 months (range: 185–1556 days), respectively, while in the HGG cohort, they were 11.0 months (range: 82–687 days) and 26.7 months (range: 296–1491 days). In conclusion, the authors suggested that achieving a more substantial antitumor response may require combining lenalidomide and radiotherapy with other chemotherapeutic agents [178].

In the same year, DeWire et al. [179] published the first data on the safety, feasibility, and early response of ribociclib (Novartis Pharmaceuticals) after radiotherapy, obtained from a phase I/II study (NCT02607124) in patients with DIPG and diffuse midline glioma (DMG) harboring H3K27M mutations. Ribociclib is a selective inhibitor of cyclin-dependent kinases (CDKs) 4 and 6, which are activated upon binding to cyclins D. The cyclin D-CDK4/6 complex regulates cell cycle progression by phosphorylating the retinoblastoma protein (pRb). Ribociclib’s mechanism of action prevents pRb from becoming hyperphosphorylated, keeping it in an active, hypophosphorylated state, thereby inducing cell cycle arrest [180]. A previous phase I study demonstrated that ribociclib induces tolerable toxicity and prolonged disease stabilization in pediatric patients with recurrent rare tumors, neuroblastoma, and tumors with defects in cell cycle pathways [181]. Within four weeks after completing radiotherapy (standard dose: 54 Gy), 10 patients (9 with DIPG and 1 with DMG) with a median age of 7.3 years (range: 3.7–19.8) received ribociclib at the RP2D (350 mg/m^2^) once daily for 21 days in 28-day cycles, for a maximum of 12 cycles. The median number of cycles administered for all 10 patients was 8 (range: 3–14), with 6 patients receiving at least 6 cycles. The most common grade 3/4 toxicities were neutropenia (90%), lymphopenia (50%), and leukopenia (70%). No patients died due to toxicity. Three patients developed grade 4 neutropenia, requiring dose reduction; in one of these cases, treatment had to be discontinued. Despite this, ribociclib therapy after radiotherapy was deemed feasible. The 1-year overall survival rate and the median overall survival for DIPG were 89% and 16.1 months (range: 10–30), respectively. The patient with diffuse midline glioma died six months after diagnosis [179].

The findings from this study prompted the initiation of two subsequent studies, both conducted by the same research team and published in the following two years. The first study [182] included a phase focused on determining the MTD and RP2D, as well as characterizing the toxicity and pharmacokinetic profiles of ribociclib and everolimus in pediatric patients. Additionally, a surgical investigation was conducted to assess the intratumoral pharmacokinetics of ribociclib at the RP2D of 350 mg/m^2^ in monotherapy (PBTC-50). In the phase I study, sixteen patients (median age: 10.3 years; range: 3.9–20.4) with recurrent or refractory malignant brain tumors were administered ribociclib at an initial dose of 120 mg/m^2^ once daily for 21 days in 28-day cycles, in combination with everolimus at an initial dose of 1.2 mg/m^2^ once daily continuously. Dose escalation followed the Rolling-6 statistical model. In the absence of disease progression or unacceptable toxicity, patients could continue treatment for up to 2 years (26 cycles). The surgical cohort included six patients (median age: 11.4 years; range: 7.2–17.1) treated with ribociclib at the pediatric RP2D (350 mg/m^2^) once daily for 7–10 days before surgery. After surgery, patients were assessed for inclusion in the phase I study, and five were assigned to dose level 1. Treatment with ribociclib and everolimus was well tolerated. However, two of the three patients treated at dose level 2 developed grade 3 hypertension and grade 4 transaminase elevation. The most common grade 3/4 toxicities were lymphopenia (30%), neutropenia (25%), and leukopenia (20%). The RP2D for ribociclib and everolimus was established at 120 mg/m^2^ for 21 days and 1.2 mg/m^2^ for 28 days, respectively. When administered alone, the pharmacokinetic profiles of both drugs in pediatric patients were consistent with previously reported data [181,183]. When combined, no impact of everolimus on the pharmacokinetics of ribociclib was observed. However, the steady-state plasma exposure to everolimus was approximately 2.5 times higher than with everolimus in monotherapy, with considerable interindividual variability. No patients showed an objective response to the ribociclib and everolimus treatment. Nonetheless, one patient with recurrent anaplastic ependymoma achieved a partial response and prolonged disease stabilization for 24 treatment cycles [182].

The second study is a phase I clinical trial (NCT03355794) assessing the safety, feasibility, and pharmacokinetic profile of ribociclib and everolimus as maintenance therapy following radiotherapy in pediatric patients with newly diagnosed DIPG, DIPG with biopsy positive for the presence of the retinoblastoma (RB) protein, and HGG [184]. Within 2–4 weeks after completing radiotherapy (standard dose: 54 Gy), nineteen patients (median age: 6.5 years; range: 2 months to 15 years) were treated with ribociclib at an initial dose of 120 mg/m^2^ once daily for 21 days in 28-day cycles, combined with everolimus at an initial dose of 1.2 mg/m^2^ once daily continuously. Each 28-day cycle could be repeated up to 24 times. One of six patients enrolled at dose level 3 (ribociclib 170 mg/m^2^ days 1–21; everolimus 1.5 mg/m^2^ days 1–28) developed a grade 3 pulmonary infection. None of the other six patients in the expansion cohort experienced further dose-limiting toxicities (DLTs) at dose level 3. Consequently, the RP2D was defined as 170 mg/m^2^ for ribociclib (days 1–21) and 1.5 mg/m^2^ for everolimus (days 1–28), with the MTD not being reached. The treatment was well tolerated. As observed in the previous study, the most common grade 3/4 toxicities were neutropenia (33%), leukopenia (17%), and lymphopenia (11%). Two patients required dose reductions—one due to a grade 3 pulmonary infection and the other for grade 3 ALT elevation associated with grade 4 hypokalemia. A third patient discontinued treatment after cycle 9 due to grade 4 cardiac toxicity. The pharmacokinetic profiles of both drugs were consistent with previously reported data in earlier studies, both in monotherapy [181,183] and in combination [182]. The median OS for patients with DIPG was 13.9 months, with a 36-month OS rate of 38.9%. Among the HGG patients, median PFS was 10.5 months. Molecular analysis of tumor tissue identified CDK4 amplification and CDKN2A/B deletion in two patients with OS of >37 and 20 months, respectively. Activation of the PI3K/mTOR pathway was observed in two patients whose tumors harbored PIK3R1 mutations and PTEN deletions, although this did not result in improved clinical outcomes [184].

Genetic studies have revealed that approximately 10% of pediatric and adult astrocytomas exhibit activating mutations in the BRAF kinase, with the BRAFV600E mutation being the most common [185]. In an effort to identify new therapeutic options for BRAF-mutated astrocytomas, Wang et al. characterized the pharmacokinetic profile of vemurafenib (Zelboraf), an oral BRAF inhibitor, in a Phase I study conducted on pediatric patients with recurrent or refractory BRAFV600E-mutated astrocytomas. Twenty-five patients with a median age of 8.8 years (range: 3.3–19.2) received vemurafenib orally twice daily in 28-day cycles. Doses were adjusted based on body surface area (m^2^) before each cycle. Nine patients were assigned to the initial dose level of 550 mg/m^2^ twice daily, equivalent to the approved adult dose of 960 mg based on an average body surface area of 1.73 m^2^. Another nine patients were assigned to dose level −1, which was 420 mg/m^2^ twice daily. Finally, six patients were enrolled in an expansion cohort to receive a formulation of the drug in crushed tablets at a dose of 550 mg/m^2^ twice daily. This study represents the most comprehensive analysis of the pharmacokinetics of vemurafenib in pediatric patients with recurrent/refractory BRAF V600E-mutant astrocytomas. The results demonstrated that the crushed tablets ensure adequate drug exposure with a relative bioavailability compared to the intact formulation estimated at 96% (95% CI: 49%–142%). Furthermore, the pharmacokinetic profiles of vemurafenib were similar to those observed in adults, although there was interindividual variability in pharmacokinetic parameters, attributable to various factors, including the effect of food, protein binding, and metabolism. According to their model, the dose equivalent to the MTD for adults is 550 mg/m^2^; therefore, the authors conclude that each patient should receive a personalized dose based on their body weight, and dose reductions may prevent excessive drug exposure in children, thereby preventing toxicity [186].

In the same year, a phase I/II study was conducted to evaluate the RP2D and pharmacokinetic parameters of veliparib, a Poly (ADP-ribose) polymerase (PARP) inhibitor, in combination with radiotherapy and temozolomide in pediatric patients with newly diagnosed DIPG. Eighteen patients aged 21 years or younger received veliparib in conjunction with radiotherapy administered at standard doses (1.8 Gy/daily fraction for a total dose of 54 Gy) over a period of 6 weeks. The initial dose of veliparib was set at 50 mg/m^2^/dose twice daily, with two dose escalations (65 and 85 mg/m^2^/dose) and one reduction (35 mg/m^2^/dose). During radiotherapy, three patients experienced dose-limiting toxicities (DLT) at dose level 3 (85 mg/m^2^/dose), including grade 2 intratumoral hemorrhage (n = 1), grade 3 maculopapular rash (n = 2), and grade 3 generalized neurological deterioration (n = 1). Therefore, the RP2D of veliparib with concomitant radiotherapy was defined as 65 mg/m^2^ twice daily. The most common toxicities during treatment with veliparib and radiotherapy were lymphopenia and neutropenia. Four weeks after radiotherapy, veliparib was administered at a dose of 25 mg/m^2^ twice daily in maintenance therapy in combination with TMZ at 135 mg/m^2^/day for 5 days every 28 days. If tolerated, the dose of TMZ could be increased to 175 and 200 mg/m^2^/day in subsequent cycles. During the maintenance phase, 2 out of 5 patients treated with 175 mg/m^2^ and 2 out of 3 patients treated with 200 mg/m^2^ experienced toxicity, leading to the cessation of intra-patient TMZ dose escalation for the remainder of the study. The most common grade 3 or higher toxicities were hematological in nature. Although the treatment was generally well tolerated, an interim analysis revealed that the 1- and 2-year OS rates were 37.2% and 5.3% with standard error of 7% and 3%, respectively. Since no improvement in survival was observed in DIPG patients, enrolment was discontinued [187].

In 2021, Van Mater et al. determined the MTD and toxicities of Palbociclib (IBRANCE, Pfizer Inc, Kenilworth, NJ, USA), a selective inhibitor of cyclin-dependent kinases (CDK) 4 and 6, in pediatric patients with progressive or refractory brain tumors [188]. Like ribociclib, Palbociclib reduces cell proliferation by blocking the progression of the cell cycle from the G1 phase to the S phase [189]. Based on the prior treatment regimens received, 21 patients were included in Stratum I (not heavily pretreated) and 14 were included in Stratum II (heavily pretreated). Palbociclib was administered orally at an initial dose of 50 mg/m^2^ for the first 21 days of a 28-day cycle, with subsequent dose escalation based on the Rolling-6 statistical model. At dose level 3 (95 mg/m^2^), 2 of the 4 enrolled patients developed grade 4 neutropenia. Therefore, an additional 9 patients were enrolled at dose level 2, and only 2/12 patients experienced a grade 3 thrombocytopenia. The MTD was established at 75 mg/m^2^ for both groups. Similar to adults [190], myelosuppression was the most frequently observed toxicity. Palbociclib treatment was considered safe, but no significant antitumor activity was observed [188].

As in 2021, with the aim of improving treatment efficacy in progressive DIPG, El-Khouly et al. proposed a therapeutic regimen capable of simultaneously targeting multiple molecular pathways involved in tumor progression. Based on the mechanism of action of the drugs, the authors assessed the safety, tolerability, and efficacy of adding erlotinib to a baseline therapy consisting of bevacizumab and irinotecan. This strategy aimed to exploit the properties of erlotinib as an inhibitor of the EGFR receptor tyrosine kinase, in synergy with the anti-angiogenic action of bevacizumab and the cytotoxic effect of irinotecan. Patients received a baseline therapy, consisting of bevacizumab (10 mg/kg) and irinotecan (125 mg/m^2^), administered intravenously in 2-week cycles for a maximum period of one year (26 cycles). Two successive cohorts of 4 and 5 patients received escalating doses of erlotinib (65 and 85 mg/m^2^), administered orally once daily. At the initial dose, one patient experienced a grade 3 acute diarrhea. No further DLTs were observed in the subsequent patients treated up to the maximum dose of 85 mg/m^2^. The median PFS was 3.2 months (range 1.0–10.9), while the median OS was 13.8 months (range 9.3–33.0), which was higher than the 10-month survival of patients with DIPG treated exclusively with radiotherapy. Therefore, erlotinib was well tolerated and a multi-target therapy could represent a promising option for patients with DIPG [191].

Fangusaro et al. [192] published a Phase I study on the use of pomalidomide, a thalidomide analogue [193], for the first time in the pediatric population. Twenty-nine patients (aged 3 to 21 years) with recurrent, progressive, and refractory CNS tumors were treated with oral pomalidomide once daily for 21 days. The initial dose was set at 1.9 mg/m^2^ once daily, with two dose escalations (2.4 mg/m^2^ and 3.6 mg/m^2^) and a reduction (1.3 mg/m^2^). The MTD and RP2D were estimated using the Rolling-6 statistical model. Pomalidomide was well tolerated. Dose-limiting toxicities of grade 3/4 were observed at dose level 3 (3.4 mg/m^2^) in 3 patients (diarrhea, thrombocytopenia, pulmonary infection, and neutropenia), and in 1 patient (thrombocytopenia) enrolled in the expansion cohort at the MTD, corresponding to dose level 2 (2.6 mg/m^2^). The most common treatment-related adverse events included lymphopenia, leukopenia, neutropenia, thrombocytopenia, anemia, fatigue, and grade 1 and 2 headaches. The patients received a median number of 2 treatment cycles (range: 1–50). At all dose levels, patients showed an increase in GZMB+ T cells (*p* = 0.034), regulatory T cells (Tregs) (*p* = 0.0017), and NKp46 levels (*p* = 0.040) between day 15 and day 21 compared to baseline, suggesting that pomalidomide induces an immune response at the serum level. One patient maintained stable disease for nine cycles, while a second patient achieved a partial response and continued therapy for over four years without recurrence. The PFS and OS at 12 months were 3.5 ± 2.4% and 28.1 ± 8.4%, respectively. Further analysis showed that patients with higher baseline levels of Tregs had significantly longer OS (*p* = 0.036) [192].

In 2022, based on data obtained from adults with recurrent glioblastoma multiforme [194,195], Metts et al. conducted a phase I study with a 3+3 design aimed at determining the MTD of irinotecan administered biweekly in combination with temozolomide and a fixed dose of bevacizumab in 28-day cycles. Twenty-six children with a median age of 12 years (range 3–22) and recurrent or refractory CNS tumors were treated with irinotecan (125–150 mg/m^2^ intravenously on days 1 and 15) and temozolomide (ranging from 75 to 200 mg/m^2^ orally on days 1–5), in combination with bevacizumab (10 mg/kg on days 1 and 15), administered in 28-day cycles for up to 24 cycles. Among the five patients treated at dose level 2, two developed grade 3 DLTs (increased ALT/AST, headache, and syncope). Three additional patients were enrolled at dose level 1 without reporting DLTs; therefore, dose level 1 was declared the MTD. Overall, the treatment was well tolerated, with positive radiographic findings: six patients completed at least 12 cycles, and two reached the full 24 cycles. With one complete response, six partial responses, and ten disease stabilizations, a global response rate of 31.8% was observed [196].

Gibson et al. characterized the pharmacokinetic profile of crizotinib, a potent tyrosine kinase inhibitor, in combination with dasatinib in a phase I study (NCT01644773) involving pediatric patients with progressive or recurrent HGG and DIPG. The first eight patients received crizotinib and dasatinib orally twice daily at initial doses of 130 and 50 mg/m^2^ for six weeks. The treatment regimen was poorly tolerated, and in the next 28 patients, the dosing was adjusted to once daily (with initial doses of 165 and 50 mg/m^2^) for four weeks. Pharmacokinetic analyses showed that crizotinib’s pharmacokinetics were affected by concomitant dasatinib use and overweight/obesity status. The mean apparent clearance (CL/F) of crizotinib was 66.7 L/h/m^2^ after a single dose, decreasing to 26.5 L/h/m^2^ at steady state when administered alone, but not when combined with dasatinib (mean 60.8 L/h/m^2^). Overweight/obese patients showed lower CL/F and apparent distribution volume (V1/F) (mean 46.2 L/h/m^2^ and 73.3 L/m^2^) compared to other patients (mean 75.5 L/h/m^2^ and 119.3 L/m^2^, *p* < 0.001). This suggests that dose adjustments might be necessary for overweight/obese patients due to a potential pharmacokinetic interaction between crizotinib and dasatinib [197].

In 2022, Bisbee et al. [198] characterized the pharmacokinetics of crenolanib, an oral PDGFR α/β and FLT3 inhibitor [199,200], for the first time in the pediatric population. Patients aged between 2.1 and 19.2 years with newly diagnosed DIPG were included in Stratum A (n = 32), and patients with recurrent, progressive, or refractory HGG were included in Stratum B (n = 23). Patients in Stratum A received crenolanib orally once daily in conjunction with radiotherapy during the first six weeks of treatment, followed by continuous daily administration for up to two years. Patients in Stratum B received continuous daily crenolanib for up to two years. In both strata, the starting dose (100 mg/m^2^) was gradually increased up to 220 mg/m^2^. Most patients received concomitant treatment (antiemetics, antibiotics, and acid secretion inhibitors) to manage neurological symptoms and reduce anticipated nausea and vomiting during crenolanib therapy. Patients on concomitant treatments, such as H2 antagonists or proton pump inhibitors, showed approximately 2 and 1.7 times lower clearance and volume, respectively (*p* < 0.0001 and *p* = 0.018). Despite a marked increase in drug exposure with concomitant acid-reducing treatment, crenolanib therapy was well tolerated, and no dose adjustments are recommended for this population [198].

Su et al. [201] conducted a phase I/II study to determine the efficacy, toxicities, and RP2D of vorinostat, an oral histone deacetylase inhibitor [166], in a pediatric population with DIPG. The phase I portion of the study involved 12 patients aged 3–21 years. During radiotherapy (1.8 Gy/daily fraction for a total dose of 54 Gy), vorinostat was administered at an initial dose of 180 mg/m^2^ once daily for 5 days a week, with an expected dose escalation to 230 mg/m^2^ once daily, adjusted according to the Rolling-6 statistical model. After completing radiotherapy, vorinostat was given at 230 mg/m^2^ once daily for up to twelve 28-day cycles. No DLTs were observed, and vorinostat at 230 mg/m^2^/day was declared the RP2D. In the expansion cohort treated at RP2D, the most common DLTs were grade 3 thrombocytopenia and grade 4 neutropenia. Although well tolerated, the regimen did not improve outcomes. One-year event-free survival was 5.85%, and overall survival was 39.2% [201].

In 2023, Monje et al. [202]evaluated the safety and tolerability of Panobinostat, a multi-histone deacetylase (HDAC) oral inhibitor, in pediatric patients with progressive DIPG and diffuse midline glioma. By inhibiting HDACs, Panobinostat modulates the expression of various genes involved in tumor suppression and cell cycle regulation. In gliomas, Panobinostat partially reverses epigenetic silencing aberrantly caused by the H3K27M mutation [203]. Initially, 19 patients with progressive DIPG were included in Stratum I, and 34 with non-progressive DIPG or H3K27M-mutant DMG were included in Stratum II. In Stratum I, panobinostat was administered orally at doses ranging from 5 to 36 mg/m^2^ three times a week for 21 days in a 28-day cycle. Dose escalation was determined using the two-stage continual reassessment method. After determining the MTD, patients in Stratum II were treated with Panobinostat orally three times a week for two weeks. In both strata, common toxicities included thrombocytopenia, neutropenia, and lymphopenia. The MTD was established at 10 mg/m^2^/dose in Stratum I and 22 mg/m^2^/dose in Stratum II, administered three times a week according to the respective dosing schedules. Pharmacokinetic analysis revealed good absorption from oral capsules, with biphasic elimination and proportional increases in C_max_ and AUC_last_ with dose, consistent with results from adult Panobinostat studies [204]. No significant clinical benefit was observed from Panobinostat. In Stratum I, patients received a median of 2 cycles (range: 1–5), with a median PFS of 2.0 months (range: 0.9–4.0 months) and a median OS of 5.2 months (range: 0.7–15.2 months). In Stratum II, patients received a median of 3 cycles (range: 1–12), with a median PFS of 4.4 months (range: 1.0–11.0 months) and a median OS of 11.8 months (range: 4.8–25.0 months) [202].

Meanwhile, Mueller et al. [205] evaluated the safety, tolerability, and distribution of MTX110 (Midatech Ltd., Cardiff, UK) administered via Convection-Enhanced Delivery (CED) in patients with DIPG who had completed radiotherapy. MTX110 is a soluble form of panobinostat that can be delivered via CED [206]. CED, or convection-enhanced delivery, is a technique that allows for the direct administration of drugs into tissues, particularly in the brain, bypassing the blood-brain barrier. This technique utilizes a pressure gradient to distribute the drug through the interstitial flow directly into the tumor tissue, offering the advantage of achieving higher concentrations at the desired site while minimizing systemic exposure and toxicity to healthy tissues [207,208]. Seven patients with a median age of 8 years (range: 5–21) received a total of 48 CED infusions of MTX110 at 7 dose levels (30–90 µM; volumes ranging from 3 mL to 2 consecutive 6 mL doses). Four patients experienced neurological DLTs (grade 3 muscle weakness, vagus nerve disorder, grade 3 gait disorder, intolerable grade 2 dysphasia). Grade 3 neutropenia was the most frequent treatment-related adverse event (4 out of 7). Median OS was 26.1 months, while PFS ranged from 4 to 14 months, with a median of 7 months [205].

In 2024, Odia et al. [209] assessed the safety and pharmacokinetics of ONC201 (dordaviprone) in pediatric patients with H3K27M mutant gliomas. ONC201, the first imipridone of its class, is a small molecule capable of effectively crossing the blood-brain barrier. It acts as a dopamine receptor D2 antagonist and a peptidase P agonist. ONC201 activates the TRAIL (TNF-related apoptosis-inducing ligand) signaling pathway independently of p53 and triggers additional mechanisms that promote tumor cell death [210]. A total of 12 patients with a median age of 9 years (range: 4–18 years) were treated with ONC201 orally, administered twice a week for two consecutive days at three dose levels (DL −1, 1, and 2: 375 mg, 500 mg, and 625 mg, respectively) in 21-day cycles. The treatment was well tolerated, with no DLT or treatment-related adverse events of grade greater than 3. A pharmacokinetic analysis was performed at DL2, revealing no drug accumulation between two successive doses. C_max_ was not increased with successive doses administered twice a week. As expected, AUC_0–48_ was higher in patients treated with ONC201 twice a week compared to those treated with a weekly regimen, with this pattern consistent across all doses (250 mg and 625 mg). However, the heterogeneity of the patient population, which included various tumor sites and a combination of recurrent and non-recurrent disease, prevented the authors from interpreting the treatment efficacy with this regimen [209].

In 2024, Krystal et al. presented the results of the first phase I dose-escalation study evaluating mebendazole in combination with bevacizumab and irinotecan for the treatment of pediatric CNS tumors. The study included 10 pediatric patients with HGG and DMG treated with mebendazole at three dose levels (50, 100, and 200 mg/kg/day) administered orally continuously for a 28-day cycle. In addition to mebendazole, patients received bevacizumab (10 mg/kg/dose) via 90-min intravenous infusion on Days 1 and 15, and irinotecan (125 mg/m^2^/dose) via 60-min intravenous infusion on the same days. If tolerated, irinotecan doses were escalated to 150 mg/m^2^/dose in cycle 2. The highest mebendazole dose tested (200 mg/kg/day) was well tolerated without DLT. As a result, the MTD was not reached, and the RP2D was established at 200 mg/kg/day. Moreover, mebendazole did not appear to increase the toxicity of bevacizumab and irinotecan. Neutropenia and grade 3/4 lymphopenia were the most common adverse events, none of which were attributed to mebendazole. The overall response rate was 33%, with two patients achieving a partial response and one patient achieving a complete response, sustained for 10 months. The median PFS and OS from the start of treatment in the study were 4.7 and 11.4 months, respectively [211].

In the same year, Johnson et al. [212] conducted the first pediatric phase I study (NCT02502708) on the use of indoximod, an oral inhibitor of the IDO (indoleamine 2,3-dioxygenase) pathway, in pediatric patients with recurrent brain tumors or newly diagnosed DIPG. Indoximod (1-methyl-D-tryptophan) is a structural analog of tryptophan that interferes with the catalytic degradation of tryptophan by the IDO1 enzyme into kynurenine, a metabolite that induces immune tolerance by suppressing effector T cells and activating regulatory T cells (Tregs). Indoximod prevents the activation of IDO-induced immunosuppressive signaling, allowing effector T cells to proliferate and attack the tumor [213]. Studies in mouse models have shown that indoximod blocks IDO-induced tolerance to apoptotic cells [214] and enhances the efficacy of chemo-immunotherapy with temozolomide [215]. The study aimed to evaluate the safety, tolerability, and antitumor activity of indoximod in combination with standard therapies. A total of 81 patients were enrolled, divided into four groups. In Group 1, 33 patients received indoximod at an initial dose of 80% of the adult RP2D, with increases to 100% and 120%, administered twice daily in 28-day cycles. Temozolomide was administered at a dose of 200 mg/m^2^/day for 5 days per cycle. In Group 2, 27 patients with recurrent/refractory glioblastoma (grade 4 gliomas as per the classification at the time of the study), ependymoma, medulloblastoma, and other rare pediatric CNS tumors were treated with indoximod at the RP2D established in Group 1. In Group 3, 14 patients received escalating doses of indoximod in combination with radiation therapy. Once the RP2D was determined, the cohort was expanded (Group 3b, n = 13) and treated with indoximod at the RP2D in combination with radiation therapy, followed by indoximod plus temozolomide. At disease progression, 18 patients from groups 1–3 chose to initiate second-line chemotherapy (cyclophosphamide 2.5 mg/kg orally once daily, with a maximum dose of 100 mg/day, and etoposide 50 mg/m^2^ orally once daily for 21 days in each 28-day cycle) while continuing treatment with indoximod. Both the combination of indoximod and TMZ (groups 1 and 2) and the combination of indoximod and radiation therapy were well tolerated. The MTD was not reached in either Group 1 or Group 3, and the 120% dose level (19.2 mg/kg per dose twice daily) was confirmed as the RP2D in the pediatric population. The most common toxicities have been attributed to the underlying disease (e.g., headache, ataxia, seizures) and chemotherapy (e.g., thrombocytopenia, anemia, neutropenia, fatigue). Grade 5 events, including cardiac arrest, respiratory failure, and stroke, occurred in three patients and were associated with tumor progression. The median OS was 13.3 months for all patients with recurrent disease and 14.4 months for patients with DIPG. Patients who achieved an objective response had a significantly longer median OS (25.2 months, range: 5.4–61.9) compared to non-responders (7.3 months, range: 0.2–62.7). Four patients remained free from active disease for over 36 months [212].

Recently, Lin et al. conducted a phase I study with a 3+3 design to evaluate the safety and efficacy of CAR T cells targeting GD2, modified with a constitutively active interleukin-7 receptor (C7R-GD2.CARTs), in the treatment of DMG with H3K27 alterations and other recurrent pediatric CNS tumors expressing GD2. Eleven patients (aged 4–18 years) underwent standard lymphodepletion with fludarabine (30 mg/m^2^ once daily for three doses) and were treated with cyclophosphamide (500 mg/m^2^ once daily for two doses), followed by one of three dose levels: GD2.CARTs (DL0) or C7R-GD2.CARTs at escalating doses (DL1 and DL2). Patients with stable or improving disease were eligible for up to three cycles of infusion at the same dose every six weeks. Cytokine release syndrome (CRS) grade 1 and tumor inflammation-associated neurotoxicity (TIAN) were common at DL1, resolving with tocilizumab and anakinra. At DL2, a grade 4 CRS occurred, which was managed with fractionated-dose infusions in subsequent patients, avoiding further severe toxicities. MRI images showed partial responses and intratumoral necrosis, with longer PFS in patients treated with C7R-GD2.CARTs compared to GD2.CARTs. Clinical responses were also observed in patients with thalamic DMG and recurrent embryonal tumors. These results demonstrate a manageable safety profile and promising responses; however, most patients experienced tumor progression despite repeated cycles. The interpretation of the results was limited by the small sample size, patient heterogeneity, and the potential effect of pseudoprogression induced by radiotherapy [216].

**Figure 5:**
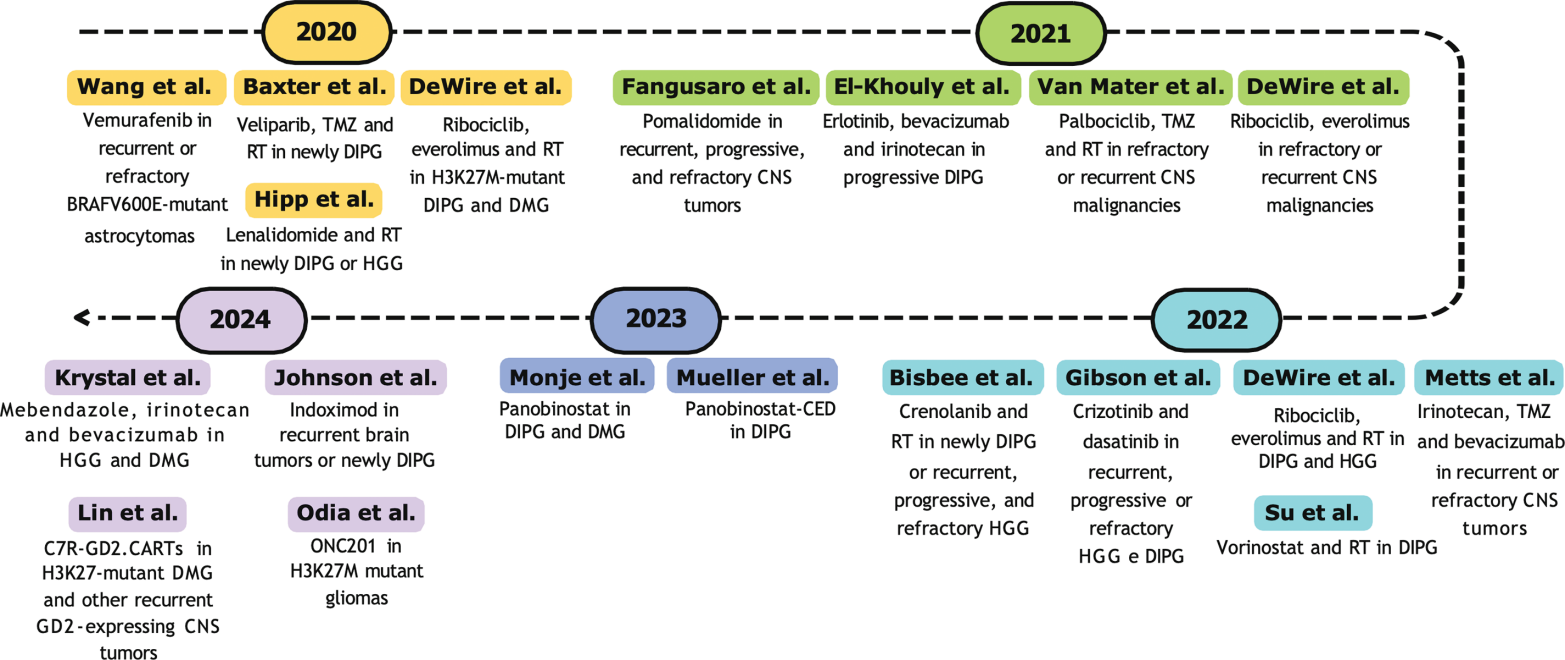
Timeline (2020–2024) of selected phase I clinical trials. RT, radiotherapy; TMZ, temozolomide; CNS, central nervous system; DIPG, diffuse intrinsic pontine glioma; HGG, high-grade glioma; DMG, diffuse midline glioma [178,179,182,184,186–188,191,192,196,197,198,201,202,205,209,211,212,216]

All studies described for the period 2020–2024 are represented in Fig. 5.

### 4.5 Ongoing Phase I Studies

Overall, 361 ongoing phase I clinical trials were identified. Of these, 60 have shared interim reports with available results. In the initial phase, duplicates were removed. Subsequently, 53 studies were excluded as they focused on neoplasms localized outside the CNS or included adult patients. Details of the 15 selected studies exclusively focused on CNS tumors in pediatric populations are provided in Table 2.

**Table 2:** Details of Phase I clinical studies focusing exclusively on central nervous system (CNS) tumors in pediatric populations. The table presents the main characteristics of each trial, the status (A = Active, but not recruiting; C = Completed; T = Terminated), the therapies, and the number of enrolled patients

Study ID	Title	Status	Therapy	n. patients
NCT02607124 [217]	A Phase I/II Study of Ribociclib, a CDK4/6 Inhibitor, Following Radiation Therapy	T	Ribociclib	11
NCT01922076 [218]	Adavosertib and Local Radiation Therapy in Treating Children with Newly Diagnosed Diffuse Intrinsic Pontine Gliomas	C	Adavosertib Radiation Therapy	46
NCT00124657 [219]	Erlotinib and Radiation Therapy in Treating Young Patients with Newly Diagnosed Glioma	C	Erlotinib hydrochloride	62
NCT00021229 [220]	Imatinib Mesylate With or Without Radiation Therapy in Treating Young Patients with Newly Diagnosed or Recurrent Glioma	T	Imatinib mesylate Radiation Therapy	85
NCT01884740 [221]	Intra-arterial Infusion of Erbitux and Bevacizumab for Relapsed/Refractory Intracranial Glioma in Patients Under 22	T	SIACI of Erbitux and Bevacizumab	13
NCT00095940 [222]	Lapatinib in Treating Young Patients with Recurrent or Refractory Central Nervous System Tumors	C	Lapatinib ditosylate	52
NCT03566199 [223]	MTX110 by Convection-Enhanced Delivery in Treating Participants with Newly Diagnosed Diffuse Intrinsic Pontine Glioma	C	MTX110	7
NCT02255461 [224]	Palbociclib Isethionate in Treating Younger Patients with Recurrent, Progressive, or Refractory Central Nervous System Tumors	T	Palbociclib isethionate	35
NCT03389802 [225]	Phase I Study of APX005M in Pediatric Central Nervous System Tumors	A	APX005M	32
NCT03451825 [226]	Phase I/II Study of Avelumab in Pediatric Cancer Participants	T	Avelumab	21
NCT03387020 [227]	Ribociclib and Everolimus in Treating Children with Recurrent or Refractory Malignant Brain Tumors	C	Everolimus Ribociclib	22
NCT00876993 [228]	Study of Irinotecan and Bevacizumab with Temozolomide in Refractory/Relapsed CNS Tumors	C	Bevacizumab Irinotecan Temozolomide	26
NCT03050450 [229]	Study of Lenalidomide with Vorinostat in Pediatric Patients with High-Grade or Progressive CNS Tumors	T	Lenalidomide Vorinostat	8
NCT02717455 [230]	Trial of Panobinostat in Children with Diffuse Intrinsic Pontine Glioma	C	LBH589	53
NCT03130959 [231]	Phase Ib /II Clinical Trial of Nivolumab Monotherapy and Nivolumab in Combination with Ipilimumab in Pediatric Subjects with High Grade Primary CNS Malignancies	T	Nivolumab Ipilimumab	166

## 5 Discussion

### 5.1 Epidemiology and Molecular Classification of Paediatric CNS Tumors

Among the most pivotal biological discoveries of the past two decades is the role of histone mutations and epigenetic modifications in the tumorigenesis of high-grade midline tumors, formerly categorized as diffuse intrinsic pontine gliomas (DIPG). Pioneering studies have revealed that these gliomas are driven by recurrent mutations in genes encoding histone 3 variants [232–234]. Among these, the substitution of lysine at position 27 with methionine (p.K27M) represents a key alteration that disrupts the function of the Polycomb Repressive Complex 2 (PRC2), the primary regulator of H3K27 trimethylation [235,236]. The resulting reduction in H3K27 trimethylation (H3K27me3) leads to widespread gene dysregulation, promoting a pro-tumorigenic environment and uncontrolled cell proliferation [235,236].

In response to these findings, the 2016 WHO Classification introduced the diagnostic category of diffuse midline glioma (DMG), H3K27M-mutant [4]. This classification was further refined in the 2021 edition, expanding the definition to diffuse midline glioma with H3K27 alteration [7]. This broader designation encompasses tumors harboring canonical H3 mutations and those exhibiting a loss of H3K27 trimethylation due to alterations in EGFR or EZHIP. Notably, recurrent mutations in EGFR and EZHIP have been implicated in PRC2 inhibition within a specific subgroup now classified under diffuse midline glioma with H3K27-alteration [237]. Despite significant advancements in molecular characterization, these tumors continue to have a grim prognosis, with a median survival of less than 12 months [202].

In addition to H3K27M-mutant DMGs, the 2021 WHO Classification introduced three additional subtypes of pediatric high-grade diffuse gliomas: H3G34R-mutant diffuse hemispheric gliomas, high-grade diffuse gliomas that are H3-wildtype and IDH-wildtype, and infant-type hemispheric gliomas. Notably, infant-type hemispheric gliomas are now recognized as a distinct entity driven by specific gene fusions and exhibit a significantly better prognosis compared to other high-grade pediatric gliomas [7]. This distinction underscores the profound clinical and therapeutic implications of the hybrid molecular classification, reinforcing the need for tailored diagnostic and treatment strategies.

A crucial factor in the substantial progress in pediatric neuro-oncology has been the evolution of clinical research, which has profoundly altered the landscape of available therapies. Over recent decades, the optimization of clinical trial designs and advancements in research methodologies have expedited the validation of innovative therapies, ensuring the development of safer and more effective experimental treatments. Furthermore, these advancements have made significant contributions to the understanding of the biological and molecular underpinnings of brain neoplasms.

This study aims to provide a comprehensive overview of the historical evolution of phase I clinical trials in pediatric oncology, with a specific focus on CNS tumors, covering the period from 1990 to 2024. The primary objective is to track advancements in phase I clinical trials dedicated solely to CNS tumors, exploring the introduction and evaluation of novel therapeutic treatments within the pediatric population. The study seeks to highlight how research strategies and therapeutic protocols have evolved over time to address the specific needs of pediatric patients, considering their biological and physiological differences from adults. Additionally, the unique challenges related to designing clinical trials for this age group are explored, including patient enrollment, difficulties in data interpretation, and risk management associated with experimental treatments. Through an extensive search of major databases—PubMed, Scopus, Embase, Web of Science, Google Scholar, EudraCT, and ClinicalTrials.gov—research identified over 1200 phase I clinical trials published between 1990 and November 2024, in addition to approximately 350 ongoing phase I trials involving pediatric patients with CNS tumors. The selection and analysis of relevant articles were conducted using Rayyan, a web-based tool that facilitated the efficient screening of studies, ensuring the inclusion of the most pertinent and impactful research in the review [76].

### 5.2 Evolution of Phase I Therapeutics

In the 1990s, studies focused exclusively on CNS tumors were relatively rare; in fact, most studies included a wide variety of solid tumors, such as bone and soft tissue tumors, in addition to CNS tumors. In the field of phase I trials, research was primarily focused on cytotoxic chemotherapy agents, including ifosfamide, carboplatin, and etoposide. In treatment protocols, chemotherapy was often combined with radiation therapy. Although this combination represented a well-established approach in pediatric oncology, it posed significant challenges, primarily due to systemic toxicity. In particular, one of the most critical aspects was hematologic toxicity, leading to myelosuppression, with subsequent neutropenia, thrombocytopenia, and anemia. These complications increased the risk of infections and hemorrhages, complicating the clinical management of pediatric patients and affecting treatment tolerability. Another crucial aspect of these studies was the limited efficacy of treatments. Despite chemotherapy agents such as paclitaxel and ifosfamide proving effective against a wide range of solid tumors, including CNS tumors, the therapeutic response in pediatric patients was often suboptimal [79,88]. This reduced efficacy was attributed to several factors, including the difficulty of crossing the blood-brain barrier. The 1990s thus ended with largely disappointing clinical results, as the addition of chemotherapy agents did not lead to a significant improvement in survival for patients with malignant brain tumors, compared to treatments based solely on radiation therapy [77,83].

Starting from the 2000s, there has been a significant shift in the therapeutic approach, with a growing interest in the use of targeted therapies, characterized by drugs with different mechanisms of action. In particular, the introduction of tyrosine kinase inhibitors (such as dasatinib and crizotinib), farnesyltransferase inhibitors (such as lonafarnib and tipifarnib), and VEGFR 1 and 2 receptor inhibitors (such as semaxanib) marked a progressive evolution towards personalized therapeutic strategies. The results obtained provided evidence that pediatric patients tolerate doses similar to those observed in adults, with considerable overlap in pharmacokinetic profiles and toxicity between the two populations [121]. From an efficacy standpoint, these agents have demonstrated promising therapeutic potential, achieving disease stabilization for periods up to six months. Despite these advancements, hematologic and gastrointestinal toxicities remained critical issues to address [107,112]. Excluding small molecules, the number of new drugs was quite limited. High-dose chemotherapy with cyclophosphamide, vincristine, and carboplatin remained the standard therapeutic option for treating medulloblastoma and high-grade glioma, although it often had limited efficacy and intolerable toxicity [94].

To minimize systemic toxicities, several preclinical studies conducted during this period provided the first evidence that administering drugs at doses lower than the MTD could be equally effective. This finding paved the way for the evaluation of metronomic therapeutic regimens for pediatric brain tumors. Metronomic chemotherapy involves the chronic administration of low doses of agents such as etoposide, temozolomide, and cyclophosphamide, either alone or in combination, at regular intervals, continuously or with very short periods of suspension. Continuous oral administration of topotecan was well tolerated up to an MTD of 0.9 mg/m^2^ per day, achieving objective responses in 15% of patients, with durations of 2.5 and 9 months [93]. In the case of temozolomide, continuous treatment for 42 days at escalating doses up to an MTD of 85 mg/m^2^ resulted in a cumulative exposure 1.5 times higher than the standard 5-day dosing regimen (120–150 mg/m^2^), with a median PFS of 7.6 weeks and a 1-year PFS rate of 22.2% [102].

With advancements in the characterization of CNS tumors and a deeper understanding of the underlying biological mechanisms, drugs targeting specific pathological processes, such as genetic alterations and abnormalities in signaling pathways, have been developed. These include small molecule EGFR inhibitors (e.g., erlotinib [129] and gefitinib [130]) and combined EGFR and ERBB2 inhibitors (e.g., lapatinib [131]). Studies have shown that these drugs have an acceptable tolerability profile in pediatric patients, even when combined with radiotherapy, with a lower incidence of dose-limiting toxicities compared to traditional cytotoxic therapies [132]. However, their efficacy is restricted to tumors with specific molecular alterations. In brainstem gliomas, patients with EGFR overexpression demonstrated a median progression-free survival of 10.1 months, which was significantly longer than that observed in EGFR-negative cases (6.3 months) [133].

During these years, clinical trials explored new drugs and multitarget therapies, including PARP inhibitors (e.g., veliparib), mTOR inhibitors, PKCβ inhibitors, and those targeting the PI3K/Akt pathways. Although these agents were generally well tolerated, their efficacy in treating gliomas and medulloblastomas was limited when used as monotherapy. However, an improvement in antitumor activity was observed when these drugs were combined either with each other or with alkylating agents [149] and anti-VEGF agents (bevacizumab) [151].

The sole exception is everolimus, which has demonstrated significant efficacy in reducing tumor volume in the treatment of subependymal giant cell astrocytoma associated with tuberous sclerosis [238]. Following the promising outcomes from clinical trials, everolimus was approved by the FDA in 2010, offering a targeted therapeutic option for patients with this rare condition, particularly in cases where the tumors were not amenable to surgical resection.

A pivotal discovery occurred in 2008, when a tandem duplication in the BRAF gene was identified in 65%–75% of low-grade pilocytic astrocytomas [239]. This alteration results in the formation of the BRAF-KIAA1549 fusion protein (f-BRAF), leading to constitutive activation of the MAPK pathway, which drives tumor growth [240]. Subsequent studies have identified additional recurrent alterations within the same pathway, including somatic mutations in BRAF, germline alterations in NF1, and changes in FGFR1/2/3, NTRK2, RAF1, ALK, and ROS1, as well as non-MAPK alterations (such as MYB and MYBL1) [241–243].

Recognizing that nearly all pLGGs are driven by single alterations in the MAPK pathway has paved the way for the development of targeted therapies, such as MEK inhibitors (selumetinib, trametinib, binimetinib) and RAF inhibitors (vemurafenib, dabrafenib). These therapies have revolutionized pLGG treatment by reducing toxicity and enhancing prognosis [244].

A recent phase II prospective study in children with untreated BRAFV600E-mutated pLGG, comparing the combination of dabrafenib-trametinib to carboplatin-vincristine, showed an objective response rate of 47% and a median progression-free survival of 20.1 months in the dabrafenib-trametinib group. This was in contrast to 11% and 7.4 months in the carboplatin-vincristine group, with significantly lower toxicity. This outcome led to the FDA’s approval of the dabrafenib-trametinib combination in 2023 as a first-line treatment for BRAFV600E-mutated pLGG, setting a new therapeutic standard for this patient subgroup [245].

However, it is important to note that, aside from everolimus for subependymal giant cell astrocytomas associated with tuberous sclerosis and the dabrafenib-trametinib combination for BRAFV600E-mutated pLGGs, the role of targeted inhibitors as first-line treatments for pLGGs remains uncertain. At present, conventional chemotherapy continues to be the most widely applied standard of care, with targeted inhibitors in first-line therapy limited to clinical trials. Although the acute toxicity profiles of these therapies are generally favorable, the long-term effects of these drugs remain poorly understood. Additionally, the phenomenon of tumor “rebound” growth in some pLGGs following the discontinuation of targeted therapy presents another challenge, with the underlying clinical and biological mechanisms still not fully elucidated [246].

In recent years, immunotherapy has become an increasingly widespread option for the treatment of pediatric cancers, including those of the central nervous system [247]. With the discovery of immune checkpoints, immune checkpoint inhibitors (ICIs) have become one of the main pillars of cancer treatment in adults [248]. The efficacy of ICIs has been correlated with the tumor mutational burden (TMB) or microsatellite instability, a surrogate marker for TMB [249]. A high number of tumor mutations leads to an increase in neoantigens, facilitating recognition by the immune system and enhancing the effectiveness of ICI therapy. Tumors characterized by a mismatch repair deficiency (MMR) exhibit an exceptionally high mutation rate, promoting the development of neoantigens and increased lymphocytic infiltration.

A recent Phase 1 clinical trial (NCT02359565) demonstrated that pediatric patients with hypermutated high-grade gliomas and mismatch repair deficiency can significantly benefit from treatment with immune checkpoint inhibitors, particularly through the use of a PD-1 inhibitor [70]. A recent prospective pediatric clinical trial with nivolumab for solid tumors with high TMB and refractory to treatment showed a 50% overall response rate, with sustained complete remissions, including patients with refractory malignant gliomas [250]. This has shifted the therapeutic approach for this small subset of patients and led to the FDA approval of pembrolizumab in 2020 for pediatric patients with recurrent solid tumors and high TMB.

One of the most recent advancements in immunotherapy is the development of chimeric antigen receptor T (CAR-T) cells. The success of CAR-T therapy directed against CD19 in high-risk hematologic cancers has paved the way for research using modified lymphocytes (primarily T or NK cells) to attack tumor cells.

Several Phase I studies have tested CAR-T cells directed against GD2, HER2, and B7H3 antigens in diffuse midline gliomas (DMG) and other recurrent/refractory pediatric brain tumors, including ependymoma and medulloblastoma [216,251]. However, these studies have revealed significant toxicities, particularly cytokine release syndrome, immune effector cell-associated neurotoxicity syndrome, and neurotoxicity related to tumor inflammation [252]. Despite these challenges, clinical and radiographic responses have been observed in some patients with treatment-resistant pediatric brain tumors, highlighting the potential of this therapeutic strategy [216,251].

In addition to CAR-T therapy, other immunotherapeutic strategies developed in recent decades include therapeutic cancer vaccines, oncolytic virus therapies, and engineered T cell receptors and cytotoxic T lymphocytes, all under investigation for various pediatric CNS tumors. These approaches represent new therapeutic frontiers aimed at improving the prognosis of pediatric brain tumors.

### 5.3 Methodological Advances in Phase I Design

To establish the safety of a treatment, most phase I studies in pediatric oncology use a 3+3 design. This design involves enrolling patients in groups of three, who receive a specific dose of the investigational drug. If none of the three experiences grade 3 or higher toxicity, the dose is considered safe, and a new group of three patients is enrolled with a higher dose. This process continues until the MTD is identified, which is the highest dose that can be administered without causing unacceptable toxicity. Once the MTD is determined, phase I studies typically progress to phase II, where the drug’s efficacy and safety at this dose are further explored in a larger patient population. The 3+3 design has the advantage of exposing a small number of participants to potentially toxic doses of experimental drugs. Being conducted on a high-risk population, these studies represent a compromise between scientific progress and patient protection, ensuring that new therapies are rigorously evaluated without compromising participant safety.

The adoption of adaptive trial models, such as the modified Continual Reassessment Method (CRM) and the Rolling 6 model, has introduced greater flexibility and precision in dose-escalation strategies while maintaining high safety standards for determining the MTD. The modified CRM is based on an ongoing statistical model that evaluates the probability of toxicity at the administered dose and predicts the safe dose based on observed toxicity events. The first group of patients is treated with an initial dose deemed safe. As patient data are collected, the statistical model is continuously updated to adjust the dose for the next group of patients. This process continues until the MTD is identified, defined as the dose level that causes acceptable toxicity, typically a severe toxicity that does not exceed 30% of patients treated at that dose. The primary goal is to determine the MTD with the fewest patients possible. The main advantage is that it reduces the risk of overdose and enhances patient safety through continuous data monitoring and corresponding dose adjustments [253].

The Rolling-6 model, first published in 2008, represents a significant modification to traditional methods, allowing the simultaneous enrollment of six patients at a given dose level. As in the CRM model, the responses of each group of six patients influence the determination of the dose for the subsequent group. However, compared to the CRM, the Rolling-6 method is more conservative: it is based on a predefined number of patients for each dose, and the dose determination for subsequent groups is based on visual and practical assessments of toxicities rather than statistical models. Additionally, because the dose is adjusted only between subsequent groups and not for individual patients, the model is relatively simpler to manage [254]. Although these studies have improved the understanding of safety profiles, the clinical efficacy of the drugs tested has often been modest, with disease stabilization as the most common best result.

Many ongoing clinical trials in pediatric oncology aim to stratify patients based on specific molecular characteristics of their tumors. These trials utilize targeted therapies or biological treatments designed to address specific molecular or genetic mechanisms that drive tumor growth, with the goal of minimizing damage to healthy tissues and enhancing treatment efficacy. The primary objective of these studies is to improve therapeutic outcomes, reduce toxicity, and increase overall patient survival. However, a key limitation is the need for advanced diagnostic testing and targeted drugs, which may not be universally available across healthcare facilities or regions.

### 5.4 Global Landscape and Collaboration Needs

Despite significant advances in pediatric neuro-oncology over the past three decades, several challenges remain. Clinical trials in pediatric neuro-oncology continue to face persistent obstacles, including the rarity and heterogeneity of pediatric brain tumors, the lack of standardized and universally accessible molecular tests, and uncertainties regarding the long-term effects of novel therapies. In the past 25 years, the establishment and growth of various consortia have transformed the pediatric neuro-oncology field, facilitating the conduct of large-scale, multicenter studies crucial for generating robust, globally relevant findings. A major challenge is the limited participation of non-U.S. research groups, which can introduce biases in understanding treatment responses across diverse genetic, environmental, and healthcare contexts. Our data show that 50 of the 60 pharmacological phase-I trials exclusively focused on paediatric CNS tumours (83%) were conducted in the United States. This concentration suggests that the availability of infrastructure, funding, and cooperative networks in the USA facilitates the design of highly targeted studies on a single disease, thereby providing a faster track to subsequent clinical phases. At the same time, the paucity of comparable studies in other regions reveals marked heterogeneity in early-stage research strategies worldwide. The dominance of US-based phase-I studies should not be viewed solely as evidence of greater operational capacity; it also underscores the need for broader, more structured international collaboration. Scientific societies such as the International Society of Paediatric Oncology (SIOP), together with North-American, European, and Asian networks, should promote shared protocols, common data platforms, and capacity-building programmes that enable centres in other parts of the world to initiate and conduct early pharmacological trials in paediatric brain tumours.

Expanding international collaboration could diversify findings and foster a more comprehensive approach to treating pediatric CNS tumors. While numerous molecular drivers of brain tumors have been identified, molecular diagnostics remain non-standardized and inconsistently accessible. This limitation restricts access to targeted therapies and hampers enrollment in clinical trials, thereby limiting opportunities for patients who might benefit from more advanced treatment approaches.

### 5.5 Ethical and Practical Considerations

In all studies included in this review, written informed consent was obtained from parents or legal guardians before participation, in accordance with institutional guidelines. For pediatric patients, it is crucial to ensure that the consent process is not merely a formal procedure but an opportunity to provide patients and their families with clear, comprehensible information. Age-appropriate communication is essential to help patients understand the disease, the treatment, and the associated risks, enabling them to be actively involved in decision-making. Strategies such as the use of visual aids, simplified explanations, and psychological support play an important role in enhancing understanding and reducing stress related to the diagnosis and treatment. A structured, transparent consent process not only strengthens the trust between medical staff, the patient, and the family but also contributes to improved adherence to treatment, emotional well-being, and overall care quality.

Adherence to therapy is a critical factor in oncology, directly affecting treatment efficacy and clinical outcomes. In children and adolescents, adherence can be influenced by numerous factors related to the drug characteristics as well as psychological, familial, and social elements. While younger children may struggle to comprehend the importance of treatment and rely entirely on parents or caregivers for consistent medication administration, adolescents, although more autonomous, may be less likely to follow medical prescriptions. The method of drug administration is another crucial consideration in pediatric patients. Younger children may be unable to swallow tablets, capsules, or pills, and the dosage provided by solid formulations may be inappropriate for them. Oral suspensions offer a viable alternative, overcoming the difficulty of swallowing pills, particularly in younger children, while ensuring more precise and measurable dosing. However, the taste of these suspensions can sometimes negatively impact patient compliance.

## 6 Conclusion

This review offers a comprehensive analysis of the evolution of Phase I trials in pediatric neuro-oncology, emphasizing the impact of emerging therapeutic strategies and evolving clinical trial methodologies. The ongoing development of adaptive trial designs, the growing personalization of treatments driven by molecular biomarkers, and the enhancement of international collaboration networks are pivotal components of research in this domain.

In conclusion, Phase I trials for pediatric CNS tumors have transitioned from traditional chemotherapy to advanced targeted therapies, reflecting a deeper and more nuanced understanding of these malignancies. Nevertheless, achieving an optimal balance between toxicity and efficacy remains a critical challenge, underscoring the necessity for continuous innovation and refinement to improve clinical outcomes in pediatric patients.

## Data Availability

The datasets generated and/or analyzed during the current study are available from the corresponding author on reasonable request.

## Abbreviations

AUC: Area Under the Plasma Concentration × Time Curve
b.i.d.: Bis In Die
CARs: Chimeric Antigen Receptors
CED: Convection-Enhanced Delivery
CRM: Continual Reassessment Method
DIPG: Diffuse Intrinsic Pontine Glioma
DLT: Dose Limiting Toxicity
DMG: Diffuse Midline Glioma
EIACDs: Enzyme-Inducing Anticonvulsant Drugs
FDA: U.S. Food and Drug Administration
IBW: Ideal Body Weight
ICI: Immune Checkpoint Inhibitors
MAPK: Mitogen-Activated Protein Kinase Kinase
MGMT: O^6^-Methylguanine-DNA Methyltransferase
MTD: Maximum Tolerated Dose
OS: Overall Survival
PFS: Progression-Free Survival
pHGGs: Pediatric High-Grade Gliomas
pLGGs: Pediatric Low-Grade Gliomas
PNET: Primitive Neuroectodermal Tumors
RP2D: Recommended Phase II Dose
CNS: Central Nervous System
TMZ: Temozolomide
VEGF: Vascular Endothelial Growth Factor
WHO: World Health Organization
